# Supplementary material for: Population Genomics, Virulence Traits, and Antimicrobial Resistance of Streptococcus suis Isolated in China
Source: Microorganisms. 2025 May 23;13(6):1197. doi: 10.3390/microorganisms13061197 (PMC12195111; doi:10.3390/microorganisms13061197)
Supplement: Supplementary file 1 [file microorganisms-13-01197-s001.zip › microorganisms-3568576-supplementary.pdf]

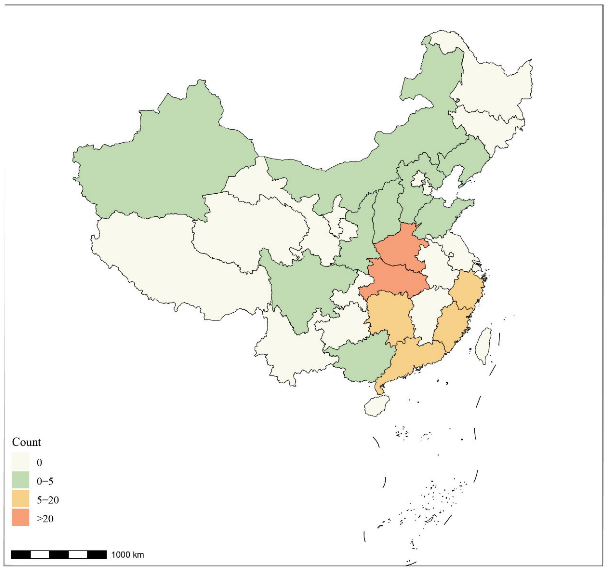

Figure S1. T The map of China showing Streptococcus suis from different provinces.

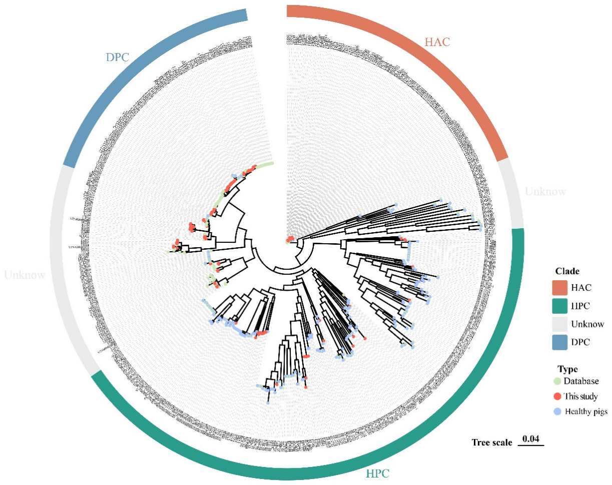

Figure S2. Phylogenetic tree of Streptococcus suis isolates of different origins. Tips in blue were obtained from the tonsils of clinically healthy pigs (n=223), tips in red for the isolates in this study (n=177), and tips in green from the database (n=240).

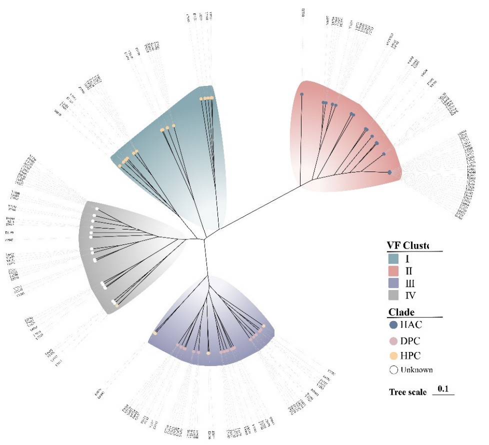

Figure S3. Phylogenetic tree (unrooted) of evolutionary clade and VAF clade. Tips are coloured according to clade, blue for HAC, red for DPC and yellow for HPC.

Table S1. The information of 177 suis isolates in this study

**Table S1 The information of 177 *suis* isolates in this study**				
Strain name	Isolation year	Province in china	Tissue source	Accession NO.
C9523	2018	China: Sichuan	Brain	SAMN44730298
C9726	2018	China: Sichuan	Lung	SAMN44730299
E1120	2019	China: Hubei	Lung	SAMN44730300
E1210	2018	China: Hubei	Lung	SAMN44730301
E1225	2019	China: Hubei	Lung	SAMN44730302
E1529	2017	China: Hubei	Lung	SAMN44730303
E1819	2017	China: Hubei	Lung	SAMN44730304
E1974	2018	China: Hubei	Lung	SAMN44730305
E1991	2018	China: Hubei	Kidney	SAMN44730306
E2126	2017	China: Hubei	Joints	SAMN44730307
E2128	2017	China: Hubei	Lung	SAMN44730308
E2309	2018	China: Hubei	Lung	SAMN44730309
E2376	2018	China: Hubei	Intestine	SAMN44730310
E2498	2017	China: Hubei	Liver	SAMN44730311
E2592	2018	China: Hubei	Brain	SAMN44730312
E2690	2017	China: Hubei	Brain	SAMN44730313
E2724	2018	China: Hubei	Lung	SAMN44730314
E2741	2018	China: Hubei	Lung	SAMN44730315
E2758	2018	China: Hubei	Lung	SAMN44730316
E2871	2018	China: Hubei	Brain	SAMN44730317
E3038	2018	China: Hubei	Lung	SAMN44730318
E3111	2018	China: Hubei	Lung	SAMN44730319
E3123	2018	China: Hubei	Lung	SAMN44730320
E3145	2018	China: Hubei	Lung	SAMN44730321
E3342	2017	China: Hubei	Brain	SAMN44730322
E3606	2019	China: Hubei	Lung	SAMN44730323
E3937	2017	China: Hubei	Lung	SAMN44730324
E3946	2018	China: Hubei	Lung	SAMN44730325

E3948	2018	China: Hubei	Lung	SAMN44730326
E4081	2018	China: Hubei	Lung	SAMN44730327
E4299	2018	China: Hubei	Lung	SAMN44730328
E4308	2018	China: Hubei	Lung	SAMN44730329
E4343	2018	China: Hubei	Brain	SAMN44730330
E4344	2017	China: Hubei	Lung	SAMN44730331
E4504	2017	China: Hubei	Brain	SAMN44730332
E4513	2019	China: Hubei	Lung	SAMN44730333
E4555	2017	China: Hubei	Lung	SAMN44730334
E4558	2018	China: Hubei	Lung	SAMN44730335
E4843	2018	China: Hubei	Effusion	SAMN44730336
E4968	2018	China: Hubei	Effusion	SAMN44730337
E5246	2018	China: Hubei	Lung	SAMN44730338
E5271	2017	China: Hubei	Brain	SAMN44730339
E5333	2017	China: Hubei	Brain	SAMN44730340
E5369	2017	China: Hubei	Lung	SAMN44730341
E5450	2018	China: Hubei	Heart	SAMN44730342
E5472	2018	China: Hubei	Brain	SAMN44730343
E5476	2017	China: Hubei	Brain	SAMN44730344
E5606	2018	China: Hubei	Lung	SAMN44730345
E5611	2018	China: Hubei	Lung	SAMN44730346
E5879	2018	China: Hubei	Lung	SAMN44730347
E6114	2017	China: Hubei	Brain	SAMN44730348
E6124	2018	China: Hubei	Lung	SAMN44730349
E6199	2017	China: Hubei	Lung	SAMN44730350
E6207	2018	China: Hubei	Lung	SAMN44730351
E6352	2019	China: Hubei	Lung	SAMN44730352
E6439	2018	China: Hubei	Lung	SAMN44730353
E6444	2019	China: Hubei	Lung	SAMN44730354
E6452	2017	China: Hubei	Lung	SAMN44730355
E6488	2017	China: Hubei	Brain	SAMN44730356
E6504	2017	China: Hubei	Lung	SAMN44730357
E6514	2018	China: Hubei	Lung	SAMN44730358
E6605	2018	China: Hubei	Lung	SAMN44730359
E6640	2018	China: Hubei	Lung	SAMN44730360
E6702	2018	China: Hubei	Brain	SAMN44730361
E6718	2017	China: Hubei	Lung	SAMN44730362
E6886	2017	China: Hubei	Brain	SAMN44730363
E6897	2018	China: Hubei	Lung	SAMN44730364
E7334	2017	China: Hubei	Lung	SAMN44730365
E7403	2017	China: Hubei	Lung	SAMN44730366
E7526	2017	China: Hubei	Brain	SAMN44730367
E7567	2017	China: Hubei	Brain	SAMN44730368
E7616	2017	China: Hubei	Brain	SAMN44730369
E7634	2018	China: Hubei	Spleen	SAMN44730370
E7657	2019	China: Hubei	Lung	SAMN44730371
E7695	2018	China: Hubei	Lung	SAMN44730372
E7716	2017	China: Hubei	Brain	SAMN44730373

E7750	2019	China: Hubei	Lung	SAMN44730374
E7885	2017	China: Hubei	Lung	SAMN44730375
E7912	2019	China: Hubei	Lung	SAMN44730376
E8036	2017	China: Hubei	Lung	SAMN44730377
E8168	2017	China: Hubei	Lung	SAMN44730378
E8229	2019	China: Hubei	Lung	SAMN44730379
E8300	2017	China: Hubei	Joints	SAMN44730380
E8420	2018	China: Hubei	Lung	SAMN44730381
E8650	2017	China: Hubei	Brain	SAMN44730382
E8772	2017	China: Hubei	Lung	SAMN44730383
E8888	2017	China: Hubei	Joints	SAMN44730384
E8894	2018	China: Hubei	Lymph node	SAMN44730385
E8996	2017	China: Hubei	Brain	SAMN44730386
E9142	2018	China: Hubei	Lung	SAMN44730387
E9349	2017	China: Hubei	Lung	SAMN44730388
E9490	2018	China: Hubei	Lung	SAMN44730389
E9696	2018	China: Hubei	Spleen	SAMN44730390
E9719	2017	China: Hubei	Lung	SAMN44730391
E9733	2017	China: Hubei	Brain	SAMN44730392
G6942	2017	China: Guangxi	Lung	SAMN44730393
J3545	2019	China: Hebei	Lung	SAMN44730394
J5817	2018	China: Hebei	Lung	SAMN44730395
J7160	2017	China: Shanxi	Lung	SAMN44730396
J9245	2017	China: Hebei	Lung	SAMN44730397
L2400	2018	China: Shandong	Lung	SAMN44730398
L2401	2018	China: Shandong	Lung	SAMN44730399
L2763	2018	China: Liaoning	Lung	SAMN44730400
L2814	2018	China: Shandong	Lung	SAMN44730401
L7545	2018	China: Liaoning	Lung	SAMN44730402
M2248	2018	China: Fujian	Lung	SAMN44730403
M6600	2017	China: Fujian	Joints	SAMN44730404
M6937	2017	China: Fujian	Lung	SAMN44730405
M7157	2017	China: Fujian	Lung	SAMN44730406
M9253	2017	China: Fujian	Joints	SAMN44730407
M9403	2018	China: Fujian	Lung	SAMN44730408
N8215	2018	China: Neimenggu	Lung	SAMN44730409
S6670	2018	China: Shaanxi	Lung	SAMN44730410
X1706	2017	China: Hunan	Lung	SAMN44730411
X2459	2019	China: Xinjiang	Lung	SAMN44730412
X3426	2017	China: Hunan	Brain	SAMN44730413
X4751	2018	China: Hunan	Lung	SAMN44730414
X4863	2018	China: Hunan	Kidney	SAMN44730415
X5705	2018	China: Hunan	Lung	SAMN44730416
X7400	2018	China: Hunan	Lung	SAMN44730417
X7828	2019	China: Xinjiang	Lung	SAMN44730418
X9177	2018	China: Hunan	Brain	SAMN44730419
X9285	2019	China: Xinjiang	Lung	SAMN44730420
X9374	2018	China: Hunan	Lung	SAMN44730421

Y1080	2018	China: Henan	Lung	SAMN44730422
Y1081	2017	China: Henan	Lung	SAMN44730423
Y1087	2018	China: Henan	Lung	SAMN44730424
Y1173	2017	China: Guangdong	Brain	SAMN44730425
Y1676	2019	China: Henan	Lung	SAMN44730426
Y1717	2018	China: Henan	Lung	SAMN44730427
Y1892	2017	China: Henan	Lung	SAMN44730428
Y1961	2018	China: Henan	Lung	SAMN44730429
Y2637	2018	China: Henan	Lung	SAMN44730430
Y2715	2017	China: Henan	Brain	SAMN44730431
Y2968	2018	China: Guangdong	Joints	SAMN44730432
Y3249	2017	China: Henan	Lung	SAMN44730433
Y3277	2017	China: Guangdong	Brain	SAMN44730434
Y3343	2018	China: Henan	Lung	SAMN44730435
Y3443	2017	China: Guangdong	Joints	SAMN44730436
Y3842	2017	China: Henan	Lung	SAMN44730437
Y4371	2018	China: Henan	Effusion	SAMN44730438
Y4934	2018	China: Henan	Lung	SAMN44730439
Y4943	2017	China: Henan	Liver	SAMN44730440
Y5009	2017	China: Henan	Brain	SAMN44730441
Y5247	2017	China: Henan	Lung	SAMN44730442
Y5411	2017	China: Henan	Lung	SAMN44730443
Y5682	2017	China: Guangdong	Joints	SAMN44730444
Y5792	2018	China: Henan	Lung	SAMN44730445
Y5923	2017	China: Henan	Lung	SAMN44730446
Y6166	2017	China: Henan	Brain	SAMN44730447
Y6197	2017	China: Henan	Lung	SAMN44730448
Y6648	2018	China: Henan	Lung	SAMN44730449
Y6784	2017	China: Henan	Lung	SAMN44730450
Y7283	2018	China: Henan	Lung	SAMN44730451
Y7354	2017	China: Henan	Lung	SAMN44730452
Y7361	2017	China: Henan	Lung	SAMN44730453
Y7511	2018	China: Henan	Lung	SAMN44730454
Y7742	2018	China: Henan	Lung	SAMN44730455
Y7767	2019	China: Henan	Lung	SAMN44730456
Y8023	2018	China: Guangdong	Brain	SAMN44730457
Y8024	2017	China: Henan	Brain	SAMN44730458
Y8131	2017	China: Henan	Lung	SAMN44730459
Y8485	2017	China: Henan	Lung	SAMN44730460
Y8500	2017	China: Henan	Lung	SAMN44730461
Y8649	2018	China: Guangdong	Lung	SAMN44730462
Y8704	2019	China: Henan	Lung	SAMN44730463
Y9217	2018	China: Guangdong	Lung	SAMN44730464
Y9239	2018	China: Henan	Lung	SAMN44730465
Y9437	2017	China: Henan	Brain	SAMN44730466
Y9446	2018	China: Henan	Lung	SAMN44730467
Y9707	2019	China: Henan	Lung	SAMN44730468
Y9931	2018	China: Henan	Liver	SAMN44730469

Z5574	2018	China: Zhejiang	Lung	SAMN44730470
Z7256	2017	China: Zhejiang	Lung	SAMN44730471
Z7590	2017	China: Zhejiang	Lung	SAMN44730472
Z9616	2019	China: Zhejiang	Lung	SAMN44730473
Z9945	2018	China: Zhejiang	Lung	SAMN44730474

Table S2. Serotypes, MLST, Clade and VF Cluster of the 177 suis used in this stud

**Table S2 Serotypes, MLST, Clade and VF Cluster of the 177 *suis* used in this study**				
Strain name	Serotype	ST sype	Clade	VF Cluster
C9523	7	ST373	unknown	IV
C9726	7	ST373	unknown	IV
E1120	3	ST117	DPC	III
E1210	3	ST117	DPC	III
E1225	NCL3	New	HPC	I
E1529	2	ST353	HAC	II
E1819	2	ST28	DPC	III
E1974	31	New	HPC	I
E1991	9	ST1307	HPC	I
E2126	2	ST25	DPC	III
E2128	2	ST7	HAC	II
E2309	2	ST1	HAC	II
E2376	2	ST28	DPC	III
E2498	2	ST242	HAC	II
E2592	2	ST353	HAC	II
E2690	2	ST353	HAC	II
E2724	18	ST76	unknown	IV
E2741	2	ST353	HAC	II
E2758	2	ST28	DPC	III
E2871	14	ST1*	HAC	II
E3038	3	ST117	DPC	III
E3111	15	New	HPC	IV
E3123	1/2	ST28	DPC	III
E3145	1/2	ST28	DPC	III
E3342	2	ST353	HAC	II
E3606	30	New	HPC	III
E3937	30	New	HPC	I
E3946	8	ST308	unknown	IV
E3948	4	ST94	unknown	IV
E4081	2	ST25	DPC	III
E4299	19	New	unknown	IV
E4308	2	ST353	HAC	II
E4343	2	ST7	HAC	II
E4344	2	ST353	HAC	II
E4504	1/2	ST28	DPC	III
E4513	2	ST7	HAC	II
E4555	7	ST29	DPC	III
E4558	1/2	ST28	DPC	III

E4843	9	New	unknown	IV
E4968	8	ST308	unknown	IV
E5246	9	ST1184	unknown	IV
E5271	2	ST353	HAC	II
E5333	2	ST353	HAC	II
E5369	2	ST7	HAC	II
E5450	2	ST242	HAC	II
E5472	3	ST117	DPC	III
E5476	2	ST353	HAC	II
E5606	3	ST117	DPC	III
E5611	8	ST308	unknown	IV
E5879	NCL17	New	HPC	III
E6114	2	ST353	HAC	II
E6124	2	ST28	DPC	III
E6199	8	ST198	unknown	IV
E6207	3	ST27	DPC	III
E6352	1	ST11	HAC	II
E6439	1/2	ST28	DPC	III
E6444	2	ST25	DPC	III
E6452	2	ST353	HAC	II
E6488	2	ST353	HAC	II
E6504	2	ST353	HAC	II
E6514	8	ST14	unknown	IV
E6605	19	ST1466	unknown	IV
E6640	4	ST1258	unknown	IV
E6702	7	ST29	DPC	III
E6718	2	ST242	HAC	II
E6886	2	ST353	HAC	II
E6897	21	ST941	unknown	IV
E7334	7	ST29	DPC	III
E7403	31	ST1911	HPC	I
E7526	2	ST353	HAC	II
E7567	2	ST353	HAC	II
E7616	1/2	ST28	DPC	III
E7634	2	ST353	HAC	II
E7657	2	ST25	DPC	III
E7695	2	ST7	HAC	II
E7716	1/2	ST28	DPC	III
E7750	3	ST117	DPC	III
E7885	21	New	HPC	I
E7912	4	ST94	unknown	IV
E8036	2	ST353	HAC	II
E8168	2	ST353	HAC	II
E8229	9	ST243	HPC	I
E8300	4	ST485	unknown	IV
E8420	16	New	HPC	I
E8650	2	ST353	HAC	II
E8772	2	ST7	HAC	II

E8888	4	ST485	unknown	IV
E8894	8	ST14*	unknown	IV
E8996	2	ST353	HAC	II
E9142	1	ST353	HAC	II
E9349	3	ST117	DPC	III
E9490	2	ST353	HAC	II
E9696	9	New	unknown	IV
E9719	3	ST27*	DPC	III
E9733	2	ST7	HAC	II
G6942	28	New	HPC	III
J3545	9	ST243	HPC	I
J5817	3	ST117	DPC	III
J7160	7	ST373	unknown	IV
J9245	2	ST28	DPC	III
L2400	9	New	HPC	I
L2401	9	New	HPC	I
L2763	7	ST373	unknown	IV
L2814	2	ST242	HAC	II
L7545	8	ST308	unknown	IV
M2248	4	ST485	unknown	IV
M6600	2	ST7	HAC	II
M6937	2	ST1	HAC	II
M7157	2	ST1	HAC	II
M9253	2	ST7	HAC	II
M9403	7	ST29	DPC	III
N8215	3	ST117	DPC	III
S6670	16	New	HPC	I
X1706	4	ST94	unknown	IV
X2459	2	ST28	DPC	III
X3426	4	ST2236	unknown	IV
X4751	3	ST108	unknown	IV
X4863	9	ST1307	HPC	I
X5705	2	ST7	HAC	II
X7400	3	ST108	unknown	IV
X7828	2	ST28	DPC	III
X9177	19	ST1466	unknown	IV
X9285	1/2	ST28	DPC	III
X9374	4	ST94	unknown	IV
Y1080	3	ST117	DPC	III
Y1081	7	ST29	DPC	III
Y1087	3	ST117	DPC	III
Y1173	9	ST1307	HPC	I
Y1676	8	ST308	unknown	IV
Y1717	1/2	ST28	DPC	III
Y1892	19	ST1466	unknown	IV
Y1961	28	New	HPC	I
Y2637	10	New	HPC	I
Y2715	2	ST1	HAC	II

Y2968	2	ST1	HAC	II
Y3249	8	ST308	unknown	IV
Y3277	2	ST7	HAC	II
Y3343	4	ST485	unknown	IV
Y3443	9	ST1307	HPC	I
Y3842	4	ST17	unknown	IV
Y4371	2	ST1	HAC	II
Y4934	2	ST1	HAC	II
Y4943	2	ST1*	HAC	II
Y5009	2	ST1*	HAC	II
Y5247	2	ST1	HAC	II
Y5411	29	New	HPC	I
Y5682	2	ST1	HAC	II
Y5792	3	ST117	DPC	III
Y5923	2	ST7	HAC	II
Y6166	2	ST1	HAC	II
Y6197	3	ST117	DPC	III
Y6648	2	ST1	HAC	II
Y6784	2	ST1	HAC	II
Y7283	4	ST485	unknown	IV
Y7354	7	ST373	unknown	IV
Y7361	9	ST243	HPC	I
Y7511	9	ST243	HPC	I
Y7742	3	ST117	DPC	III
Y7767	7	ST29	DPC	III
Y8023	9	ST243	HPC	I
Y8024	18	ST941	unknown	IV
Y8131	3	ST1004	DPC	III
Y8485	3	ST117	DPC	III
Y8500	2	ST7	HAC	II
Y8649	3	ST117	DPC	III
Y8704	9	ST243	HPC	I
Y9217	9	ST1307	HPC	I
Y9239	29	New	HPC	I
Y9437	2	ST1	HAC	II
Y9446	2	ST353	HAC	II
Y9707	5	New	HPC	III
Y9931	2	ST1	HAC	II
Z5574	3	ST117	DPC	III
Z7256	17	New	unknown	IV
Z7590	9	ST243	HPC	I
Z9616	8	ST14	unknown	IV
Z9945	4	New	unknown	IV

Table S3 The matrix of the presence or absence of VAFs in 177 suis genomes

Table S3 The matrix of the presence or absence of VAFs in 177 *suis* genomes

Strain name	VF	MRP	epf	sly	1910HK	1910HR	ABC1	ABC2	AgaR2	ArcD	ArgR	BgaC	ClpP	ClpX	CodY	CovR	DivIVA	DnaJ	DnaK	EF-Tu	FBA	FBPS	Fhb1	Fhb2
C9523	IV	0	1	1	1	1	1	1	1	1	1	1	1	1	1	1	1	1	1	1	1	1	0	0
C9726	IV	0	1	1	1	1	1	1	1	1	1	1	1	1	1	1	1	1	1	1	1	1	0	0
E1120	III	0	0	0	0	0	1	1	1	1	1	1	1	1	1	1	1	1	1	1	1	1	1	0
E1210	III	0	0	0	0	0	1	1	1	1	1	1	1	1	1	1	1	1	1	1	1	1	0	0
E1225	I	0	0	0	0	0	1	0	0	1	1	1	1	1	1	1	1	1	1	1	1	1	0	0
E1529	II	1	1	1	1	1	1	1	1	1	1	1	1	1	1	1	1	1	1	1	1	1	0	1
E1819	III	0	0	0	0	0	1	1	1	1	1	1	1	1	1	1	1	1	1	1	1	1	1	0
E1974	I	0	0	0	1	1	1	1	1	1	1	1	1	1	1	1	1	1	1	1	1	1	1	0
E1991	I	0	0	0	1	1	1	1	1	1	1	1	1	1	1	1	1	1	1	1	1	1	0	0
E2126	III	0	0	0	0	0	1	1	1	1	1	1	1	1	1	1	1	1	1	1	1	1	0	0
E2128	II	1	1	1	1	1	1	1	1	1	1	1	1	1	1	1	1	1	1	1	1	1	0	1
E2309	II	1	1	1	1	1	1	1	1	1	1	1	1	1	1	1	1	1	1	1	1	1	1	1
E2376	III	0	0	0	0	0	1	1	1	1	1	1	1	1	1	1	1	1	1	1	1	1	0	0
E2498	II	1	1	1	1	1	1	1	1	1	1	1	1	1	1	1	1	1	1	1	1	1	1	1
E2592	II	1	1	1	1	1	1	1	1	1	1	1	1	1	1	1	1	1	1	1	1	1	1	1
E2690	II	1	1	1	1	1	1	1	1	1	1	1	1	1	1	1	1	1	1	1	1	1	0	1
E2724	IV	0	1	1	1	1	1	1	1	1	1	1	1	1	1	1	1	1	1	1	1	1	0	0
E2741	II	1	1	1	1	1	1	1	1	1	1	1	1	1	1	1	1	1	1	1	1	1	1	1
E2758	III	0	0	0	0	0	1	1	1	1	1	1	1	1	1	1	1	1	1	1	1	1	0	0
E2871	II	1	1	1	1	1	1	1	1	1	1	1	1	1	1	1	1	1	1	1	1	1	0	1
E3038	III	0	0	0	0	0	1	1	1	1	1	1	1	1	1	1	1	1	1	1	1	1	0	0
E3111	IV	0	0	1	1	1	1	1	1	1	1	1	1	1	1	1	1	1	1	1	1	1	0	0
E3123	III	0	0	0	0	0	1	1	1	1	1	1	1	1	1	1	1	1	1	1	1	1	0	0
E3145	III	0	0	0	0	0	1	1	1	1	1	1	1	1	1	1	1	1	1	1	1	1	0	0
E3342	II	1	1	1	1	1	1	1	1	1	1	1	1	1	1	1	1	1	1	1	1	1	0	1
E3606	III	0	0	0	0	0	1	1	1	1	1	1	1	1	1	1	1	1	1	1	1	1	0	0
E3937	I	0	0	0	0	0	1	0	1	0	1	1	1	1	1	1	1	1	1	1	1	1	0	0
E3946	IV	1	0	1	1	1	1	1	1	1	1	1	1	1	1	0	1	1	1	1	1	1	0	0
E3948	IV	0	1	1	1	1	1	1	1	1	1	1	1	1	1	1	1	1	1	1	1	1	0	0
E4081	III	0	0	0	0	0	1	1	1	1	1	1	1	1	1	1	1	1	1	1	1	1	0	0
E4299	IV	0	1	1	1	1	1	1	1	1	1	1	1	1	1	1	1	1	1	1	1	1	0	0
E4308	II	1	1	1	1	1	1	1	1	1	1	1	1	1	1	1	1	1	1	1	1	1	1	1
E4343	II	1	1	1	1	1	1	1	1	1	1	1	1	1	1	1	1	1	1	1	1	1	1	0
E4344	II	1	1	1	1	1	1	1	1	1	1	1	1	1	1	1	1	1	1	1	1	1	0	1
E4504	III	0	0	0	0	0	1	1	1	1	1	1	1	1	1	1	1	1	1	1	1	1	0	0
E4513	II	1	1	1	1	1	1	1	1	1	1	1	1	1	1	1	1	1	1	1	1	1	0	1
E4555	III	0	0	0	0	0	1	1	1	1	1	1	1	1	1	1	1	1	1	1	1	1	0	0
E4558	III	0	0	0	0	0	1	1	1	1	1	1	1	1	1	1	1	1	1	1	1	1	0	0
E4843	IV	0	1	1	1	1	1	1	1	1	1	1	1	1	1	1	1	1	1	1	1	1	0	0
E4968	IV	1	0	1	1	1	1	1	1	1	1	1	1	1	1	0	1	1	1	1	1	1	0	0
E5246	IV	0	1	1	1	1	1	1	1	1	1	1	1	1	1	1	1	1	1	1	1	1	0	0
E5271	II	1	1	1	1	1	1	1	1	1	1	1	1	1	1	1	1	1	1	1	1	1	0	1
E5333	II	1	1	1	1	1	1	1	1	1	1	1	1	1	1	1	1	1	1	1	1	1	0	1
E5369	II	1	1	1	1	1	1	1	1	1	1	1	1	1	1	1	1	1	1	1	1	1	0	1
E5450	II	1	1	1	1	1	1	1	1	1	1	1	1	1	1	1	1	1	1	1	1	1	1	1
E5472	III	0	0	0	0	0	1	1	1	1	1	1	1	1	1	1	1	1	1	1	1	1	1	0
E5476	II	1	1	1	1	1	1	1	1	1	1	1	1	1	1	1	1	1	1	1	1	1	0	1
E5606	III	0	0	0	0	0	1	1	1	1	1	1	1	1	1	1	1	1	1	1	1	1	1	0
E5611	IV	1	0	1	1	1	1	1	1	1	1	1	1	1	1	0	1	1	1	1	1	1	0	0

E5879	III	0	0	1	0	0	1	1	1	1	1	1	1	1	1	1	1	1	1	1	1	1	0	0
E6114	II	1	1	1	1	1	1	1	1	1	1	1	1	1	1	1	1	1	1	1	1	1	0	1
E6124	III	0	0	0	0	0	1	1	1	1	1	1	1	1	1	1	1	1	1	1	1	1	1	0
E6199	IV	0	0	1	1	1	1	1	1	1	1	1	1	1	1	1	1	1	1	1	1	1	1	0
E6207	III	0	0	0	0	0	1	1	1	1	1	1	1	1	1	1	1	1	1	1	1	1	1	0
E6352	II	1	0	1	1	1	1	1	1	1	1	1	1	1	1	1	1	1	1	1	1	1	1	0
E6439	III	0	0	0	0	0	1	1	1	1	1	1	1	1	1	1	1	1	1	1	1	1	0	0
E6444	III	0	0	0	0	0	1	1	1	1	1	1	1	1	1	1	1	1	1	1	1	1	0	0
E6452	II	1	1	1	1	1	1	1	1	1	1	1	1	1	1	1	1	1	1	1	1	1	0	1
E6488	II	1	1	1	1	1	1	1	1	1	1	1	1	1	1	1	1	1	1	1	1	1	0	1
E6504	II	1	1	1	1	1	1	1	1	1	1	1	1	1	1	1	1	1	1	1	1	1	0	1
E6514	IV	0	1	1	1	1	1	1	1	1	1	1	1	1	1	1	1	1	1	1	1	1	0	0
E6605	IV	0	1	1	1	1	1	1	1	1	1	1	1	1	1	1	1	1	1	1	1	1	1	0
E6640	IV	0	0	1	1	1	1	1	1	1	1	1	1	1	1	1	1	1	1	1	1	1	0	0
E6702	III	1	0	0	0	0	1	1	1	1	1	1	1	1	1	1	1	1	1	1	1	1	1	0
E6718	II	1	1	1	1	1	1	1	1	1	1	1	1	1	1	1	1	1	1	1	1	1	0	1
E6886	II	1	1	1	1	1	1	1	1	1	1	1	1	1	1	1	1	1	1	1	1	1	0	1
E6897	IV	0	1	1	1	1	1	1	1	1	1	1	1	1	1	1	1	1	1	1	1	1	1	0
E7334	III	0	0	0	0	0	1	1	1	1	1	1	1	1	1	1	1	1	1	1	1	1	0	0
E7403	I	0	0	0	1	1	1	1	1	1	1	1	1	1	1	1	1	1	1	1	1	1	0	0
E7526	II	1	1	1	1	1	1	1	1	1	1	1	1	1	1	1	1	1	1	1	1	1	0	1
E7567	II	1	1	1	1	1	1	1	1	1	1	1	1	1	1	1	1	1	1	1	1	1	0	1
E7616	III	0	0	0	0	0	1	1	1	1	1	1	1	1	1	1	1	1	1	1	1	1	1	0
E7634	II	1	1	1	1	1	1	1	1	1	1	1	1	1	1	1	1	1	1	1	1	1	0	1
E7657	III	0	0	0	0	0	1	1	1	1	1	1	1	1	1	1	1	1	1	1	1	1	0	0
E7695	II	1	1	1	1	1	1	1	1	1	1	1	1	1	1	1	1	1	1	1	1	1	1	1
E7716	III	0	0	0	0	0	1	1	1	1	1	1	1	1	1	1	1	1	1	1	1	1	1	0
E7750	III	0	0	0	0	0	1	1	1	1	1	1	1	1	1	1	1	1	1	1	1	1	0	0
E7885	I	0	0	0	1	1	1	1	1	1	1	1	1	1	1	1	1	1	1	1	1	1	0	0
E7912	IV	0	1	1	1	1	1	1	1	1	1	1	1	1	1	1	1	1	1	1	1	1	1	0
E8036	II	1	1	1	1	1	1	1	1	1	1	1	1	1	1	1	1	1	1	1	1	1	0	1
E8168	II	1	1	1	1	1	1	1	1	1	1	1	1	1	1	1	1	1	1	1	1	1	1	1
E8229	I	0	0	0	1	1	1	1	1	1	1	1	1	1	1	1	1	1	1	1	1	1	1	0
E8300	IV	0	1	1	1	1	1	1	1	1	1	1	1	1	1	1	1	1	1	1	1	1	0	0
E8420	I	0	0	0	1	1	1	1	1	1	1	1	1	1	1	1	1	1	1	1	1	1	0	0
E8650	II	1	1	1	1	1	1	1	1	1	1	1	1	1	1	1	1	1	1	1	1	1	0	1
E8772	II	1	1	1	1	1	1	1	1	1	1	1	1	1	1	1	1	1	1	1	1	1	0	1
E8888	IV	0	1	1	1	1	1	1	1	1	1	1	1	1	1	1	1	1	1	1	1	1	1	0
E8894	IV	0	1	1	1	1	1	1	1	1	1	1	1	1	1	1	1	1	1	1	1	1	0	0
E8996	II	1	1	1	1	1	1	1	1	1	1	1	1	1	1	1	1	1	1	1	1	1	0	1
E9142	II	1	1	1	1	1	1	1	1	1	1	1	1	1	1	1	1	1	1	1	1	1	1	0
E9349	III	0	0	0	0	0	1	1	1	1	1	1	1	1	1	1	1	1	1	1	1	1	0	0
E9490	II	1	1	1	1	1	1	1	1	1	1	1	1	1	1	1	1	1	1	1	1	1	0	1
E9696	IV	0	1	1	1	1	1	1	1	1	1	1	1	1	1	1	1	1	1	1	1	1	0	0
E9719	III	0	0	0	0	0	1	1	1	1	1	1	1	1	1	1	1	1	1	1	1	1	0	0
E9733	II	1	1	1	1	1	1	1	1	1	1	1	1	1	1	1	1	1	1	1	1	1	0	1
G6942	III	0	0	1	0	0	1	1	1	1	1	1	1	1	1	1	1	1	1	1	1	1	0	0
J3545	I	0	0	0	1	1	1	1	1	1	1	1	1	1	1	1	1	1	1	1	1	1	1	0
J5817	III	0	0	0	0	0	1	1	1	1	1	1	1	1	1	1	1	1	1	1	1	1	0	0
J7160	IV	0	1	0	1	1	1	1	1	1	1	1	1	1	1	1	1	1	1	1	1	1	0	0
J9245	III	0	0	0	0	0	1	1	1	1	1	1	1	1	1	1	1	1	1	1	1	1	1	0
L2400	I	0	0	0	1	1	1	1	1	1	1	1	1	1	1	1	1	1	1	1	1	1	0	0
L2401	I	0	0	0	1	1	1	1	1	1	1	1	1	1	1	1	1	1	1	1	1	1	0	0

L2763	IV	0	1	1	1	1	1	1	1	1	1	1	1	1	1	1	1	1	1	1	1	1	0	0
L2814	II	1	1	1	1	1	1	1	1	1	1	1	1	1	1	1	1	1	1	1	1	1	0	1
L7545	IV	1	0	1	1	1	1	1	1	1	1	1	1	1	1	0	1	1	1	1	1	1	0	0
M2248	IV	0	1	1	1	1	1	1	1	1	1	1	1	1	1	1	1	1	1	1	1	1	0	0
M6600	II	1	1	1	1	1	1	1	1	1	1	1	1	1	1	1	1	1	1	1	1	1	0	1
M6937	II	1	1	1	1	1	1	1	1	1	1	1	1	1	1	1	1	1	1	1	1	1	1	1
M7157	II	1	1	1	1	1	1	1	1	1	1	1	1	1	1	1	1	1	1	1	1	1	0	1
M9253	II	1	1	1	1	1	1	1	1	1	1	1	1	1	1	1	1	1	1	1	1	1	1	1
M9403	III	1	0	0	0	0	1	1	1	1	1	1	1	1	1	1	1	1	1	1	1	1	0	0
N8215	III	0	0	0	0	0	1	1	1	1	1	1	1	1	1	1	1	1	1	1	1	1	0	0
S6670	I	0	0	0	1	1	1	1	1	1	1	1	1	1	1	1	1	1	1	1	1	1	1	0
X1706	IV	1	1	1	1	1	1	1	1	1	1	1	1	1	1	1	1	1	1	1	1	1	1	0
X2459	III	0	1	1	1	1	1	1	1	1	1	1	1	1	1	1	1	1	1	1	1	1	0	0
X3426	IV	0	0	0	0	0	1	1	1	1	1	1	1	1	1	1	1	1	1	1	1	1	1	0
X4751	IV	0	0	1	1	1	1	1	1	1	1	1	1	1	1	1	1	1	1	1	1	1	0	0
X4863	I	0	1	1	1	1	1	1	1	1	1	1	1	1	1	1	1	1	1	1	1	1	0	0
X5705	II	0	0	0	1	1	1	1	1	1	1	1	1	1	1	1	1	1	1	1	1	1	0	0
X7400	IV	1	1	1	1	1	1	1	1	1	1	1	1	1	1	1	1	1	1	1	1	1	0	1
X7828	III	0	1	1	1	1	1	1	1	1	1	1	1	1	1	1	1	1	1	1	1	1	1	0
X9177	IV	0	0	0	0	0	1	1	1	1	1	1	1	1	1	1	1	1	1	1	1	1	1	0
X9285	III	0	1	1	1	1	1	1	1	1	1	1	1	1	1	1	1	1	1	1	1	1	1	0
X9374	IV	0	0	0	0	0	1	1	1	1	1	1	1	1	1	1	1	1	1	1	1	1	1	0
Y1080	III	0	1	1	1	1	1	1	1	1	1	1	1	1	1	1	1	1	1	1	1	1	0	0
Y1081	III	0	0	0	0	0	1	1	1	1	1	1	1	1	1	1	1	1	1	1	1	1	1	0
Y1087	III	1	0	0	0	0	1	1	1	1	1	1	1	1	1	1	1	1	1	1	1	1	0	0
Y1173	I	0	0	0	0	0	1	1	1	1	1	1	1	1	1	1	1	1	1	1	1	1	1	0
Y1676	IV	0	0	0	1	1	1	1	1	1	1	1	1	1	1	1	1	1	1	1	1	1	0	0
Y1717	III	1	0	1	1	1	1	1	1	1	1	1	1	1	1	0	1	1	1	1	1	1	0	0
Y1892	IV	0	0	0	0	0	1	1	1	1	1	1	1	1	1	1	1	1	1	1	1	1	1	0
Y1961	I	0	1	1	1	1	1	1	1	1	1	1	1	1	1	1	1	1	1	1	1	1	1	0
Y2637	I	0	0	0	1	1	1	1	1	1	1	1	1	1	1	1	1	1	1	1	1	1	0	0
Y2715	II	0	0	0	1	1	1	1	1	1	1	1	1	1	1	1	1	1	1	1	1	1	0	0
Y2968	II	1	1	1	1	1	1	1	1	1	1	1	1	1	1	1	1	1	1	1	1	1	1	0
Y3249	IV	1	1	1	1	1	1	1	1	1	1	1	1	1	1	1	1	1	1	1	1	1	0	1
Y3277	II	1	0	1	1	1	1	1	1	1	1	1	1	1	1	0	1	1	1	1	1	1	0	0
Y3343	IV	1	1	1	1	1	1	1	1	1	1	1	1	1	1	1	1	1	1	1	1	1	0	1
Y3443	I	0	1	1	1	1	1	1	1	1	1	1	1	1	1	1	1	1	1	1	1	1	1	0
Y3842	IV	0	0	0	1	1	1	1	1	1	1	1	1	1	1	1	1	1	1	1	1	1	0	0
Y4371	II	0	0	1	1	1	1	1	1	1	1	1	1	1	1	1	1	1	1	1	1	1	0	0
Y4934	II	1	1	1	1	1	1	1	1	1	1	1	1	1	1	1	1	1	1	1	1	1	1	0
Y4943	II	1	1	1	1	1	1	1	1	1	1	1	1	1	1	1	1	1	1	1	1	1	1	0
Y5009	II	1	1	1	1	1	1	1	1	1	1	1	1	1	1	1	1	1	1	1	1	1	1	1
Y5247	II	1	1	1	1	1	1	1	1	1	1	1	1	1	1	1	1	1	1	1	1	1	0	1
Y5411	I	1	1	1	1	1	1	1	1	1	1	1	1	1	1	1	1	1	1	1	1	1	1	0
Y5682	II	0	0	0	0	0	1	0	1	0	1	1	1	1	1	1	1	1	1	1	1	1	0	0
Y5792	III	1	1	1	1	1	1	1	1	1	1	1	1	1	1	1	1	1	1	1	1	1	0	1
Y5923	II	0	0	0	0	0	1	1	1	1	1	1	1	1	1	1	1	1	1	1	1	1	1	0
Y6166	II	1	1	1	1	1	1	1	1	1	1	1	1	1	1	1	1	1	1	1	1	1	0	1
Y6197	III	1	1	1	1	1	1	1	1	1	1	1	1	1	1	1	1	1	1	1	1	1	1	0
Y6648	II	0	0	0	0	0	1	1	1	1	1	1	1	1	1	1	1	1	1	1	1	1	0	0
Y6784	II	1	1	1	1	1	1	1	1	1	1	1	1	1	1	1	1	1	1	1	1	1	0	1
Y7283	IV	1	1	1	1	1	1	1	1	1	1	1	1	1	1	1	1	1	1	1	1	1	0	1
Y7354	IV	0	1	1	1	1	1	1	1	1	1	1	1	1	1	1	1	1	1	1	1	1	0	0

Y7361	I	0	1	0	1	1	1	1	1	1	1	1	1	1	1	1	1	1	1	1	1	1	0	0
Y7511	I	0	0	0	1	1	1	1	1	1	1	1	1	1	1	1	1	1	1	1	1	1	1	0
Y7742	III	0	0	0	1	1	1	1	1	1	1	1	1	1	1	1	1	1	1	1	1	1	1	0
Y7767	III	0	0	0	0	0	1	1	1	1	1	1	1	1	1	1	1	1	1	1	1	1	1	0
Y8023	I	1	0	0	0	0	1	1	1	1	1	1	1	1	1	1	1	1	1	1	1	1	0	0
Y8024	IV	0	0	0	1	1	1	1	1	1	1	1	1	1	1	1	1	1	1	1	1	1	1	0
Y8131	III	0	1	1	1	1	1	1	1	1	1	1	1	1	1	1	1	1	1	1	1	1	0	0
Y8485	III	0	0	0	0	0	1	1	1	1	1	1	1	1	1	1	1	1	1	1	1	1	1	0
Y8500	II	0	0	0	0	0	1	1	1	1	1	1	1	1	1	1	1	1	1	1	1	1	0	0
Y8649	III	1	1	1	1	1	1	1	1	1	1	1	1	1	1	1	1	1	1	1	1	1	0	1
Y8704	I	0	0	0	0	0	1	1	1	1	1	1	1	1	1	1	1	1	1	1	1	1	0	0
Y9217	I	0	0	0	1	1	1	1	1	1	1	1	1	1	1	1	1	1	1	1	1	1	1	0
Y9239	I	0	0	0	1	1	1	1	1	1	1	1	1	1	1	1	1	1	1	1	1	1	0	0
Y9437	II	0	0	0	0	0	1	0	1	1	1	1	1	1	1	1	1	1	1	1	1	1	0	0
Y9446	II	1	1	1	1	1	1	1	1	1	1	1	1	1	1	1	1	1	1	1	1	1	1	0
Y9707	III	1	1	1	1	1	1	1	1	1	1	1	1	1	1	1	1	1	1	1	1	1	1	1
Y9931	II	0	0	0	0	0	1	1	1	1	1	1	1	1	1	1	1	1	1	1	1	1	0	0
Z5574	III	1	1	1	1	1	1	1	1	1	1	1	1	1	1	1	1	1	1	1	1	1	1	0
Z7256	IV	1	0	0	0	0	1	1	1	1	1	1	1	1	1	1	1	1	1	1	1	1	0	0
Z7590	I	0	1	1	1	1	1	1	1	1	1	1	1	1	1	1	1	1	1	1	1	1	0	0
Z9616	IV	0	0	0	1	1	1	1	1	1	1	1	1	1	1	1	1	1	1	1	1	1	1	0
Z9945	IV	0	1	1	1	1	1	1	1	1	1	1	1	1	1	1	1	1	1	1	1	1	0	0

Fhbp	FlpS	GAPDH	GdpP	GidA	GlnA	GntR	GroEL	GrpE	HAM1	HP1083	HP1538	HP1717	Hhly3	HrcA	HtpsC	IMPDH_1	IMPDH_2	IdeS	IgdE	KAR	LDH	LuxS	MsmK	NeuB	NisK	NisR	OppA
0	1	1	1	1	1	1	1	1	1	1	1	1	0	1	1	1	1	0	0	1	1	1	1	0	0	0	1
0	1	1	1	1	1	1	1	1	1	1	1	1	0	1	1	1	1	0	0	1	1	1	1	0	0	0	1
0	1	1	1	1	1	1	1	1	1	1	1	1	0	1	1	1	1	1	0	1	1	1	1	0	0	0	1
0	1	1	1	1	1	1	1	1	1	1	1	1	0	1	1	1	1	1	0	1	1	1	1	0	0	0	1
0	1	1	1	1	1	1	1	1	1	1	1	1	0	1	0	1	1	0	0	1	1	1	1	0	0	0	0
1	1	1	1	1	1	1	1	1	1	1	1	1	0	1	1	1	1	1	1	1	1	1	1	1	0	0	1
0	1	1	1	1	1	1	1	1	1	1	1	1	0	1	1	1	1	0	0	1	1	1	1	1	0	0	1
0	1	1	1	1	1	1	1	1	1	1	1	1	1	1	0	1	1	0	0	1	1	1	1	0	1	1	0
0	1	1	1	1	1	1	1	1	1	1	1	1	0	1	0	1	1	0	0	1	1	1	1	0	0	0	1
0	1	1	1	1	1	1	1	1	1	1	1	1	0	1	1	1	1	0	0	1	1	1	1	1	0	0	1
1	1	1	1	1	1	1	1	1	1	1	1	1	0	1	1	1	1	1	1	1	1	1	1	1	0	0	1
1	1	1	1	1	1	1	1	1	1	1	1	1	0	1	1	1	1	1	1	1	1	1	1	1	0	0	1
0	1	1	1	1	1	1	1	1	1	1	1	1	0	1	1	1	1	0	0	1	1	1	1	1	0	0	1
1	1	1	1	1	1	1	1	1	1	1	1	1	0	1	1	1	1	1	1	1	1	1	1	1	0	0	1
1	1	1	1	1	1	1	1	1	1	1	1	1	0	1	1	1	1	1	1	1	1	1	1	1	0	0	1
1	1	1	1	1	1	1	1	1	1	1	1	1	0	1	1	1	1	1	1	1	1	1	1	1	0	0	1
0	1	1	1	1	1	1	1	1	1	1	1	1	0	1	1	1	1	0	0	1	1	1	1	0	0	0	0
1	1	1	1	1	1	1	1	1	1	1	1	1	0	1	1	1	1	1	1	1	1	1	1	1	0	0	1
0	1	1	1	1	1	1	1	1	1	1	1	1	0	1	1	1	1	0	0	1	1	1	1	1	0	0	1
1	1	1	1	1	1	1	1	1	1	1	1	1	0	1	1	1	1	1	1	1	1	1	1	1	0	0	1
0	1	1	1	1	1	1	1	1	1	1	1	1	0	1	1	1	1	0	0	1	1	1	1	0	0	0	1
0	1	1	1	1	1	1	1	1	1	1	1	1	0	1	1	1	1	0	0	1	1	1	1	0	0	0	1
0	1	1	1	1	1	1	1	1	1	1	1	1	0	1	1	1	1	0	0	1	1	1	1	1	0	0	1
0	1	1	1	1	1	1	1	1	1	1	1	1	0	1	1	1	1	0	0	1	1	1	1	1	0	0	1
1	1	1	1	1	1	1	1	1	1	1	1	1	0	1	1	1	1	1	1	1	1	1	1	1	0	0	1
0	1	1	1	1	1	1	1	1	1	1	1	1	0	1	1	1	1	0	0	1	1	1	1	0	0	0	0
0	1	1	1	1	1	1	1	0	1	1	1	1	0	1	0	1	1	0	0	1	1	1	1	0	0	0	0
0	1	1	1	1	1	1	1	1	1	1	1	1	0	1	1	1	1	0	0	1	1	1	1	0	0	0	1

0	1	1	1	1	1	1	1	1	1	1	1	1	0	1	1	1	1	0	0	1	1	1	1	0	0	0	1
0	1	1	1	1	1	1	1	1	1	1	1	1	0	1	1	1	1	0	0	1	1	1	1	1	0	0	1
0	1	1	1	1	1	1	1	1	1	1	1	1	0	1	1	1	1	0	0	1	1	1	1	0	0	0	0
1	1	1	1	1	1	1	1	1	1	1	1	1	0	1	1	1	1	1	1	1	1	1	1	1	0	0	1
1	1	1	1	1	1	1	1	1	1	1	1	1	0	1	1	1	1	1	1	1	1	1	1	1	0	0	1
1	1	1	1	1	1	1	1	1	1	1	1	1	0	1	1	1	1	1	1	1	1	1	1	1	0	0	1
0	1	1	1	1	1	1	1	1	1	1	1	1	0	1	1	1	1	0	0	1	1	1	1	1	0	0	1
1	1	1	1	1	1	1	1	1	1	1	1	1	0	1	1	1	1	1	1	1	1	1	1	1	0	0	1
0	1	1	1	1	1	1	1	1	1	1	1	1	0	1	1	1	1	0	0	1	1	1	1	0	0	0	1
0	1	1	1	1	1	1	1	1	1	1	1	1	0	1	1	1	1	0	0	1	1	1	1	1	0	0	1
0	1	1	1	1	1	1	1	1	1	1	1	1	0	1	1	1	1	0	0	1	1	1	1	0	0	0	1
0	1	1	1	1	1	1	1	1	1	1	1	1	0	1	1	1	1	0	0	1	1	1	1	0	0	0	1
0	1	1	1	1	1	1	1	1	1	1	1	1	0	1	1	1	1	0	0	1	1	1	1	0	0	0	1
1	1	1	1	1	1	1	1	1	1	1	1	1	0	1	1	1	1	1	1	1	1	1	1	1	0	0	1
1	1	1	1	1	1	1	1	1	1	1	1	1	0	1	1	1	1	1	1	1	1	1	1	1	0	0	1
1	1	1	1	1	1	1	1	1	1	1	1	1	0	1	1	1	1	1	1	1	1	1	1	1	0	0	1
1	1	1	1	1	1	1	1	1	1	1	1	1	0	1	1	1	1	1	1	1	1	1	1	1	0	0	1
0	1	1	1	1	1	1	1	1	1	1	1	1	0	1	1	1	1	0	0	1	1	1	1	0	0	0	1
1	1	1	1	1	1	1	1	1	1	1	1	1	0	1	1	1	1	1	1	1	1	1	1	1	0	0	1
0	1	1	1	1	1	1	1	1	1	1	1	1	0	1	1	1	1	1	0	1	1	1	1	0	0	0	1
0	1	1	1	1	1	1	1	1	1	1	1	1	0	1	1	1	1	0	0	1	1	1	1	0	0	0	1
0	1	1	1	1	1	1	1	1	1	1	1	1	0	1	1	1	1	0	0	1	1	1	1	0	0	0	1
1	1	1	1	1	1	1	1	1	1	1	1	1	0	1	1	1	1	1	1	1	1	1	1	1	0	0	1
0	1	1	1	1	1	1	1	1	1	1	1	1	0	1	1	1	1	0	0	1	1	1	1	1	0	0	1
0	1	1	1	1	1	1	1	1	1	1	1	1	0	1	1	1	1	0	0	1	1	1	1	0	0	0	1
0	1	1	1	1	1	1	1	1	1	1	1	1	0	1	1	1	1	0	0	1	1	1	1	0	0	0	1
0	1	1	1	1	1	1	1	1	1	1	1	1	0	1	1	1	1	0	1	1	1	1	1	1	0	0	1
0	1	1	1	1	1	1	1	1	1	1	1	1	0	1	1	1	1	0	0	1	1	1	1	1	0	0	1
0	1	1	1	1	1	1	1	1	1	1	1	1	0	1	1	1	1	0	0	1	1	1	1	1	0	0	1
1	1	1	1	1	1	1	1	1	1	1	1	1	0	1	1	1	1	1	1	1	1	1	1	1	0	0	1
1	1	1	1	1	1	1	1	1	1	1	1	1	0	1	1	1	1	1	1	1	1	1	1	1	0	0	1
1	1	1	1	1	1	1	1	1	1	1	1	1	0	1	1	1	1	1	1	1	1	1	1	1	0	0	1
0	1	1	1	1	1	1	1	1	1	1	1	1	0	1	1	1	1	1	0	1	1	1	1	0	0	0	1
0	1	1	1	1	1	1	1	1	1	1	1	1	0	1	1	1	1	0	0	1	1	1	1	0	0	0	0
0	1	1	1	1	1	1	1	1	1	1	1	1	0	1	1	1	1	1	0	1	1	1	1	0	0	0	1
0	1	1	1	1	1	1	1	1	1	1	1	1	0	1	1	1	1	0	0	1	1	1	1	0	0	0	1
1	1	1	1	1	1	1	1	1	1	1	1	1	0	1	1	1	1	1	1	1	1	1	1	1	0	0	1
1	1	1	1	1	1	1	1	1	1	1	1	1	0	1	1	1	1	1	1	1	1	1	1	1	0	0	0
0	1	1	1	1	1	1	1	1	1	1	1	1	0	1	1	1	1	0	0	1	1	1	1	0	0	0	0
0	1	1	1	1	1	1	1	1	1	1	1	1	0	1	1	1	1	0	0	1	1	1	1	0	0	0	1
0	1	1	1	1	1	1	1	1	1	1	1	1	1	1	0	1	1	0	0	1	1	1	1	0	0	0	1
1	1	1	1	1	1	1	1	1	1	1	1	1	0	1	1	1	1	1	1	1	1	1	1	1	0	0	1
1	1	1	1	1	1	1	1	1	1	1	1	1	0	1	1	1	1	1	1	1	1	1	1	1	0	0	1
0	1	1	1	1	1	1	1	1	1	1	1	1	0	1	1	1	1	0	0	1	1	1	1	1	0	0	1
1	1	1	1	1	1	1	1	1	1	1	1	1	0	1	1	1	1	1	1	1	1	1	1	1	0	0	1
0	1	1	1	1	1	1	1	1	1	1	1	1	0	1	1	1	1	0	0	1	1	1	1	1	0	0	1
1	1	1	1	1	1	1	1	1	1	1	1	1	0	1	1	1	1	1	0	1	1	1	1	1	0	0	1
0	1	1	1	1	1	1	1	1	1	1	1	1	0	1	1	1	1	0	0	1	1	1	1	1	0	0	1
0	1	1	1	1	1	1	1	1	1	1	1	1	0	1	1	1	1	1	0	1	1	1	1	0	0	0	1
0	1	1	1	1	1	1	1	0	1	1	1	1	0	1	0	1	1	0	0	1	1	1	1	0	0	0	0
0	1	1	1	1	1	1	1	1	1	1	1	1	0	1	1	1	1	0	0	1	1	1	1	0	0	0	1
1	1	1	1	1	1	1	1	1	1	1	1	1	0	1	1	1	1	1	1	1	1	1	1	1	0	0	1
1	1	1	1	1	1	1	1	1	1	1	1	1	0	1	1	1	1	1	1	1	1	1	1	1	0	0	1

0	1	1	1	1	1	1	1	1	1	1	1	1	0	1	1	1	1	1	0	1	1	1	1	0	0	0	1
0	1	1	1	1	1	1	1	1	1	1	1	1	0	1	1	1	1	0	0	1	1	1	1	0	0	0	1
0	1	1	1	1	1	1	1	1	1	1	1	1	0	1	0	1	1	0	0	1	1	1	1	1	0	0	1
1	1	1	1	1	1	1	1	1	1	1	1	1	0	1	1	1	1	1	1	1	1	1	1	1	0	0	1
1	1	1	1	1	1	1	1	1	1	1	1	1	0	1	1	1	1	1	1	1	1	1	1	1	0	0	1
0	1	1	1	1	1	1	1	1	1	1	1	1	0	1	1	1	1	0	0	1	1	1	1	0	0	0	1
0	1	1	1	1	1	1	1	1	1	1	1	1	0	1	1	1	1	1	0	1	1	1	1	0	0	0	1
1	1	1	1	1	1	1	1	1	1	1	1	1	0	1	1	1	1	1	1	1	1	1	1	1	0	0	1
1	1	1	1	1	1	1	1	1	1	1	1	1	0	1	1	1	1	1	1	1	1	1	1	1	0	0	1
0	1	1	1	1	1	1	1	1	1	1	1	1	0	1	1	1	1	1	0	1	1	1	1	0	0	0	1
1	1	1	1	1	1	1	1	1	1	1	1	1	0	1	1	1	1	1	1	1	1	1	1	1	0	0	1
0	1	1	1	1	1	1	1	1	1	1	1	1	0	1	1	1	1	0	0	1	1	1	1	0	0	0	1
0	1	1	1	1	1	1	1	1	1	1	1	1	0	1	1	1	1	0	0	1	1	1	1	0	0	0	1
1	1	1	1	1	1	1	1	1	1	1	1	1	0	1	1	1	1	1	1	1	1	1	1	1	0	0	1
0	1	1	1	1	1	1	1	1	1	1	1	1	0	1	1	1	1	0	0	1	1	1	1	0	0	0	1
0	1	1	1	1	1	1	1	1	1	1	1	1	0	1	1	1	1	1	0	1	1	1	1	0	0	0	1
0	1	1	1	1	1	1	1	1	1	1	1	1	0	1	1	1	1	0	0	1	1	1	1	0	0	0	1
0	1	1	1	1	1	1	1	1	1	1	1	1	0	1	1	1	1	0	0	1	1	1	1	0	0	0	1
0	1	1	1	1	1	1	1	1	1	1	1	1	0	1	1	1	1	0	0	1	1	1	1	1	0	0	1
1	1	1	1	1	1	1	1	1	1	1	1	1	0	1	1	1	1	0	0	1	1	1	1	0	0	0	1
1	1	1	1	1	1	1	1	1	1	1	1	1	0	1	1	1	1	0	0	1	1	1	1	0	0	0	1
0	1	1	1	1	1	1	1	1	1	1	1	1	0	1	1	1	1	0	0	1	1	1	1	0	0	0	1
1	1	1	1	1	1	1	1	1	1	1	1	1	0	1	1	1	1	1	1	1	1	1	1	1	0	0	1
0	1	1	1	1	1	1	1	1	1	1	1	1	0	1	1	1	1	0	1	1	1	1	1	0	0	0	1
0	1	1	1	1	1	1	1	1	1	1	1	1	0	1	1	1	1	0	0	1	1	1	1	0	0	0	1
1	1	1	1	1	1	1	1	1	1	1	1	1	0	1	1	1	1	1	1	1	1	1	1	1	0	0	1
1	1	1	1	1	1	1	1	1	1	1	1	1	0	1	1	1	1	1	1	1	1	1	1	1	0	0	1
1	1	1	1	1	1	1	1	1	1	1	1	1	0	1	1	1	1	1	1	1	1	1	1	1	0	0	1
1	1	1	1	1	1	1	1	1	1	1	1	1	0	1	1	1	1	1	1	1	1	1	1	1	0	0	1
0	1	1	1	1	1	1	1	1	1	1	1	1	0	1	1	1	1	0	0	1	1	1	1	0	0	0	1
0	1	1	1	1	1	1	1	1	1	1	1	1	0	1	1	1	1	0	0	1	1	1	1	0	0	0	1
0	1	1	1	1	0	1	1	1	1	1	1	1	1	1	1	1	1	0	0	1	1	1	1	1	0	0	1
1	1	1	1	1	1	1	1	1	1	1	1	1	1	1	1	1	1	1	1	1	1	1	1	1	1	1	1
0	1	1	1	1	1	1	1	1	1	1	1	1	0	1	1	1	1	0	0	1	1	1	1	0	0	0	1
0	1	1	1	1	1	1	1	1	1	1	1	1	0	1	1	1	1	0	0	1	1	1	1	1	0	0	1
0	1	1	1	1	1	1	1	1	1	1	1	1	0	1	1	1	1	1	0	1	1	1	1	0	0	0	1
0	1	1	1	1	1	1	1	1	1	1	1	1	0	1	1	1	1	0	0	1	1	1	1	0	0	0	1
0	1	1	1	1	1	1	1	1	1	1	1	1	0	1	0	1	1	0	0	1	1	1	1	0	0	0	1
1	1	1	1	1	1	1	1	1	1	1	1	1	0	1	1	1	1	1	0	1	1	1	1	1	0	0	1
0	1	1	1	1	1	1	1	1	1	1	1	1	0	1	1	1	1	0	0	1	1	1	1	0	0	0	1
0	1	1	1	1	1	1	1	1	1	1	1	1	0	1	1	1	1	0	0	1	1	1	1	1	0	0	0
0	1	1	1	1	1	1	1	1	1	1	1	1	0	1	1	1	1	0	0	1	1	1	1	0	0	0	1
0	1	1	1	1	1	1	1	1	1	1	1	1	0	1	1	1	1	0	0	1	1	1	1	1	0	0	1
0	1	1	1	1	1	1	1	1	1	1	1	1	0	1	1	1	1	0	0	1	1	1	1	0	0	0	1
0	1	1	1	1	1	1	1	1	1	1	1	1	0	1	1	1	1	1	0	1	1	1	1	0	0	0	1
0	1	1	1	1	1	1	1	1	1	1	1	1	0	1	1	1	1	0	0	1	1	1	1	0	0	0	1
0	1	1	1	1	1	1	1	1	1	1	1	1	0	1	1	1	1	0	0	1	1	1	1	0	0	0	1
0	1	1	1	1	1	1	1	1	1	1	1	1	0	1	0	1	1	0	0	1	1	1	1	0	0	0	1
0	1	1	1	1	1	1	1	1	1	1	1	1	0	1	1	1	1	0	0	1	1	1	1	0	0	0	1
0	1	1	1	1	1	1	1	1	1	1	1	1	0	1	1	1	1	0	0	1	1	1	1	1	0	0	0
0	1	1	1	1	1	1	1	1	1	1	1	1	0	1	1	1	1	0	0	1	1	1	1	0	0	0	1
0	1	1	1	1	1	1	1	1	1	1	1	1	0	1	1	1	1	0	0	1	1	1	1	0	0	0	1
0	1	1	1	1	1	1	1	1	1	1	1	1	1	1	0	1	1	0	0	1	1	1	1	0	0	0	1

1	1	1	1	1	1	1	1	1	1	1	1	1	0	1	1	1	1	0	1	1	1	1	1	1	0	0	1
1	1	1	1	1	1	1	1	1	1	1	1	1	0	1	1	1	1	1	1	1	1	1	1	1	0	0	1
0	1	1	1	1	1	1	1	1	1	1	1	1	0	1	1	1	1	0	1	1	1	1	1	0	0	0	1
1	1	1	1	1	1	1	1	1	1	1	1	1	0	1	1	1	1	1	1	1	1	1	1	1	0	0	1
0	1	1	1	1	1	1	1	1	1	1	1	1	0	1	1	1	1	0	0	1	1	1	1	0	0	0	1
0	1	1	1	1	1	1	1	1	1	1	1	1	0	1	0	1	1	0	0	1	1	1	1	0	0	0	1
0	1	1	1	1	1	1	1	1	1	1	1	1	1	1	1	1	1	1	0	1	1	1	1	0	1	1	1
1	1	1	1	1	1	1	1	1	1	1	1	1	0	1	1	1	1	0	1	1	1	1	1	1	0	0	1
1	1	1	1	1	1	1	1	1	1	1	1	1	0	1	1	1	1	0	1	1	1	1	1	1	0	0	1
1	1	1	1	1	1	1	1	1	1	1	1	1	0	1	1	1	1	1	1	1	1	1	1	1	0	0	1
1	1	1	1	1	1	1	1	1	1	1	1	1	0	1	1	1	1	1	1	1	1	1	1	1	0	0	1
1	1	1	1	1	1	1	1	1	1	1	1	1	0	1	1	1	1	0	1	1	1	1	1	1	0	0	0
0	1	1	1	1	1	1	1	1	1	1	1	1	0	1	0	1	1	0	0	1	1	1	1	0	0	0	1
1	1	1	1	1	1	1	1	1	1	1	1	1	0	1	1	1	1	1	1	1	1	1	1	1	0	0	1
0	1	1	1	1	1	1	1	1	1	1	1	1	0	1	1	1	1	1	0	1	1	1	1	0	0	0	1
1	1	1	1	1	1	1	1	1	1	1	1	1	0	1	1	1	1	1	1	1	1	1	1	1	0	0	1
1	1	1	1	1	1	1	1	1	1	1	1	1	0	1	1	1	1	0	1	1	1	1	1	1	0	0	1
0	1	1	1	1	1	1	1	1	1	1	1	1	0	1	1	1	1	0	0	1	1	1	1	0	0	0	1
1	1	1	1	1	1	1	1	1	1	1	1	1	1	1	1	1	1	1	1	1	1	1	1	1	0	0	1
1	1	1	1	1	1	1	1	1	1	1	1	1	0	1	1	1	1	1	1	1	1	1	1	1	0	0	1
0	1	1	1	1	1	1	1	1	1	1	1	1	0	1	1	1	1	0	0	1	1	1	1	0	0	0	1
0	1	1	1	1	1	1	1	1	1	1	1	1	0	1	1	1	1	0	0	1	1	1	1	0	0	0	1
0	1	1	1	1	1	1	1	1	1	1	1	1	0	1	1	1	1	1	0	1	1	1	1	0	0	0	1
0	1	1	1	1	1	1	1	1	1	1	1	1	0	1	1	1	1	1	0	1	1	1	1	0	0	0	1
0	1	1	1	1	1	1	1	1	1	1	1	1	0	1	1	1	1	1	0	1	1	1	1	0	0	0	1
0	1	1	1	1	1	1	1	1	1	1	1	1	0	1	1	1	1	0	0	1	1	1	1	0	0	0	1
0	1	1	1	1	1	1	1	1	1	1	1	1	0	1	1	1	1	1	0	1	1	1	1	0	0	0	0
0	1	1	1	1	1	1	1	1	1	1	1	1	0	1	1	1	1	0	0	1	1	1	1	0	0	0	1
0	1	1	1	1	1	1	1	1	1	1	1	1	0	1	1	1	1	1	0	1	1	1	1	0	0	0	1
0	1	1	1	1	1	1	1	1	1	1	1	1	0	1	1	1	1	1	0	1	1	1	1	0	0	0	1
1	1	1	1	1	1	1	1	1	1	1	1	1	0	1	1	1	1	1	1	1	1	1	1	1	0	0	1
0	1	1	1	1	1	1	1	1	1	1	1	1	0	1	1	1	1	1	1	1	1	1	1	0	0	0	1
0	1	1	1	1	1	1	1	1	1	1	1	1	0	1	1	1	1	1	0	1	1	1	1	0	0	0	1
0	1	1	1	1	1	1	1	1	1	1	1	1	0	1	0	1	1	0	0	1	1	1	1	0	0	0	0
0	1	1	1	1	1	1	1	1	1	1	1	1	0	1	0	1	1	0	0	1	1	1	1	0	0	0	1
1	1	1	1	1	1	1	1	1	1	1	1	1	0	1	1	1	1	0	1	1	1	1	1	1	0	0	1
1	1	1	1	1	1	1	1	1	1	1	1	1	0	1	1	1	1	1	1	1	1	1	1	1	0	0	1
0	1	1	1	1	1	1	1	1	1	1	1	1	0	1	1	1	1	0	0	1	1	1	1	0	0	0	1
1	1	1	1	1	1	1	1	1	1	1	1	1	0	1	1	1	1	1	1	1	1	1	1	1	0	0	1
0	1	1	1	1	1	1	1	1	1	1	1	1	0	1	1	1	1	1	0	1	1	1	1	0	0	0	0
0	1	1	1	1	1	1	1	1	1	1	1	1	0	1	1	1	1	0	0	1	1	1	1	0	0	0	1
0	1	1	1	1	1	1	1	1	1	1	1	1	0	1	1	1	1	0	0	1	1	1	1	0	0	0	1
0	1	1	1	1	1	1	1	1	1	1	1	1	0	1	1	1	1	1	0	1	1	1	1	0	0	0	1

PDH	PGK	PGM	PepF	PnuC	RelA	RelQ	Rex	RggTR	SP1	Sbp1	Sbp2	SntA	SsPep	Ssa	Ssads	SspA	SspB	Stk	Stp	Tran	VraSR	Zmp	abpb	adhE	cbp40omp40	ciaH	ciaR
1	1	1	1	0	1	1	1	0	1	0	0	1	1	1	0	1	1	1	1	1	1	1	1	1	0	1	1
1	1	1	1	0	1	1	1	0	1	0	0	1	1	1	0	1	1	1	1	1	1	1	1	1	0	1	1
1	1	1	1	0	1	1	1	0	1	0	0	1	1	1	0	1	1	1	1	0	1	1	1	1	0	1	1
1	1	1	1	0	1	1	1	0	0	0	0	1	1	1	0	1	1	1	1	0	1	1	1	1	0	1	1
1	1	1	1	0	1	1	1	1	1	0	0	1	1	1	0	1	1	1	1	0	1	0	0	1	0	1	1
1	1	1	1	1	1	1	1	1	1	1	1	1	1	1	0	1	1	1	1	1	1	1	1	1	1	1	1
1	1	1	1	0	1	1	1	0	0	0	0	1	1	1	0	1	1	1	1	0	1	1	1	1	0	1	1
1	1	1	1	0	1	1	1	0	1	0	0	1	0	1	0	1	1	1	1	0	1	1	0	1	0	1	1

1	1	1	1	0	1	1	1	0	1	0	0	1	0	1	0	1	1	1	1	0	1	1	0	1	0	1	1
1	1	1	1	0	1	1	1	0	1	0	0	1	1	1	0	1	1	1	1	0	1	1	1	1	0	1	1
1	1	1	1	1	1	1	1	1	1	1	1	1	1	1	0	1	1	1	1	1	1	1	1	1	1	1	1
1	1	1	1	1	1	1	1	1	0	1	1	1	1	1	0	1	1	1	1	1	1	1	1	1	1	1	1
1	1	1	1	0	1	1	1	0	0	0	0	1	1	1	0	1	1	1	1	0	1	1	1	1	0	1	1
1	1	1	1	1	1	1	1	1	1	1	1	1	1	1	0	1	1	1	1	1	1	1	1	1	1	1	1
1	1	1	1	1	1	1	1	1	1	1	1	1	1	1	0	1	1	1	1	1	1	1	1	1	1	1	1
1	1	1	1	1	1	1	1	1	1	1	1	1	1	1	0	1	1	1	1	1	1	1	1	1	1	1	1
1	1	1	1	0	1	1	1	0	0	0	0	1	1	1	0	1	1	1	1	1	1	1	1	1	0	1	1
1	1	1	1	1	1	1	1	1	1	1	1	1	1	1	0	1	1	1	1	1	1	1	1	1	1	1	1
1	1	1	1	0	1	1	1	0	0	0	0	1	1	1	0	1	1	1	1	0	1	1	1	1	0	1	1
1	1	1	1	1	1	1	1	1	1	1	1	1	1	1	0	1	1	1	1	1	1	1	1	1	1	1	1
1	1	1	1	0	1	1	1	0	1	0	0	1	1	1	0	1	1	1	1	0	1	1	1	1	0	1	1
1	1	1	1	0	1	1	1	1	1	1	0	1	1	1	1	1	1	1	1	0	1	1	0	1	0	1	1
1	1	1	1	0	1	1	1	0	0	0	0	1	1	1	0	1	1	1	1	0	1	1	1	1	0	1	1
1	1	1	1	0	1	1	1	0	0	0	0	1	1	1	0	1	1	1	1	0	1	1	1	1	0	1	1
1	1	1	1	1	1	1	1	1	1	1	1	1	1	1	0	1	1	1	1	1	1	1	1	1	1	1	1
1	1	1	1	0	1	1	1	0	0	0	0	1	0	1	0	1	1	1	1	0	1	1	1	1	0	1	1
1	1	1	1	0	1	1	1	0	1	0	0	1	0	0	0	1	1	1	1	0	1	0	0	1	0	1	1
1	1	1	1	0	1	1	1	0	1	0	0	1	1	1	1	1	1	1	1	0	1	1	1	1	0	1	1
1	1	1	1	0	1	1	1	0	1	0	0	1	1	1	0	1	1	1	1	1	1	1	1	1	0	1	1
1	1	1	1	0	1	1	1	0	1	0	0	1	1	1	0	1	1	1	1	0	1	1	1	1	0	1	1
1	1	1	1	0	1	1	1	0	0	0	0	1	1	1	0	1	1	1	1	1	1	1	1	1	0	1	1
1	1	1	1	1	1	1	1	1	1	1	1	1	1	1	0	1	1	1	1	1	1	1	1	1	1	1	1
1	1	1	1	1	1	1	1	1	1	1	1	1	1	1	0	1	1	1	1	1	1	1	1	1	1	1	1
1	1	1	1	1	1	1	1	1	1	1	1	1	1	1	0	1	1	1	1	1	1	1	1	1	1	1	1
1	1	1	1	0	1	1	1	0	1	0	0	1	1	1	0	1	1	1	1	0	1	1	1	1	0	1	1
1	1	1	1	1	1	1	1	1	1	1	1	1	1	1	0	1	1	1	1	1	1	1	1	1	1	1	1
1	1	1	1	0	1	1	1	0	1	0	0	1	1	1	0	1	1	1	1	0	1	1	1	1	0	1	1
1	1	1	1	0	1	1	1	0	1	0	0	1	1	1	0	1	1	1	1	0	1	1	1	1	0	1	1
1	1	1	1	0	1	1	1	0	1	0	0	1	1	1	0	1	1	1	1	1	1	1	1	1	0	1	1
1	1	1	1	0	1	1	1	0	1	0	0	1	1	1	0	1	1	1	1	0	1	1	1	1	0	1	1
1	1	1	1	0	1	1	1	0	1	0	0	1	1	1	0	1	1	1	1	1	1	1	1	1	0	1	1
1	1	1	1	1	1	1	1	1	1	1	1	1	1	1	0	1	1	1	1	1	1	1	1	1	1	1	1
1	1	1	1	1	1	1	1	1	1	1	1	1	1	1	0	1	1	1	1	1	1	1	1	1	1	1	1
1	1	1	1	1	1	1	1	1	1	1	1	1	1	1	0	1	1	1	1	1	1	1	1	1	1	1	1
1	1	1	1	1	1	1	1	1	1	1	1	1	1	1	0	1	1	1	1	1	1	1	1	1	1	1	1
1	1	1	1	0	1	1	1	0	1	0	0	1	1	1	0	1	1	1	1	0	1	1	1	1	0	1	1
1	1	1	1	1	1	1	1	1	1	1	1	1	1	1	0	1	1	1	1	1	1	1	1	1	1	1	1
1	1	1	1	0	1	1	1	0	1	0	0	1	1	1	0	1	1	1	1	0	1	1	1	1	0	1	1
1	1	1	1	0	1	1	1	0	1	0	0	1	1	1	1	1	1	1	1	0	1	1	1	1	0	1	1
1	1	1	1	0	1	1	1	0	1	1	0	1	1	1	0	1	1	1	1	0	1	1	1	1	0	1	1
1	1	1	1	1	1	1	1	1	1	1	1	1	1	1	0	1	1	1	1	1	1	1	1	1	1	1	1
1	1	1	1	0	1	1	1	0	1	0	0	1	1	1	0	1	1	1	1	0	1	1	1	1	0	1	1
1	1	1	1	0	1	1	1	0	1	1	0	1	1	1	0	1	1	1	1	1	1	1	1	1	0	1	1
1	1	1	1	0	1	1	1	0	1	0	0	1	1	1	0	1	1	1	1	0	1	1	1	1	0	1	1
1	1	1	1	1	1	1	1	1	0	1	1	1	1	1	0	1	1	1	1	1	1	1	1	1	1	1	1
1	1	1	1	0	1	1	1	0	0	0	0	1	1	1	0	1	1	1	1	0	1	1	1	1	0	1	1
1	1	1	1	0	1	1	1	0	1	0	0	1	1	1	0	1	1	1	1	0	1	1	1	1	0	1	1
1	1	1	1	1	1	1	1	1	1	1	1	1	1	1	0	1	1	1	1	1	1	1	1	1	1	1	1
1	1	1	1	1	1	1	1	1	1	1	1	1	1	1	0	1	1	1	1	1	1	1	1	1	1	1	1
1	1	1	1	1	1	1	1	1	1	1	1	1	1	1	0	1	1	1	1	1	1	1	1	1	1	1	1
1	1	1	1	0	1	1	1	0	1	1	0	1	1	1	0	1	1	1	1	1	1	1	1	1	0	1	1

1	1	1	1	0	1	1	1	0	0	0	0	1	1	1	0	1	1	1	1	1	1	1	1	1	0	1	1
1	1	1	1	0	1	1	1	1	1	1	0	1	1	1	1	1	1	1	1	1	1	1	1	1	0	1	1
1	1	1	1	0	1	1	1	0	1	0	0	1	1	1	0	1	1	1	1	0	1	1	1	1	0	1	1
1	1	1	1	1	1	1	1	1	1	1	1	1	1	1	0	1	1	1	1	1	1	1	1	1	1	1	1
1	1	1	1	1	1	1	1	1	1	1	1	1	1	1	0	1	1	1	1	1	1	1	1	1	1	1	1
1	1	1	1	0	1	1	1	0	0	0	0	1	1	1	0	1	1	1	1	1	1	1	1	1	0	1	1
1	1	1	1	0	1	1	1	0	1	0	0	1	1	1	0	1	1	1	1	0	1	1	1	1	0	1	1
1	1	1	1	0	1	1	1	0	0	0	0	1	0	0	1	1	1	1	1	0	1	1	0	1	0	1	1
1	1	1	1	1	1	1	1	1	1	1	1	1	1	1	0	1	1	1	1	1	1	1	1	1	1	1	1
1	1	1	1	1	1	1	1	1	1	1	1	1	1	1	0	1	1	1	1	1	1	1	1	1	1	1	1
1	1	1	1	0	1	1	1	0	0	0	0	1	1	1	0	1	1	1	1	0	1	1	1	1	0	1	1
1	1	1	1	1	1	1	1	1	1	1	1	1	1	1	0	1	1	1	1	1	1	1	1	1	1	1	1
1	1	1	1	0	1	1	1	0	1	0	0	1	1	1	0	1	1	1	1	0	1	1	1	1	0	1	1
1	1	1	1	1	1	1	1	1	1	1	1	1	1	1	0	1	1	1	1	1	1	1	1	1	1	1	1
1	1	1	1	0	1	1	1	0	0	0	0	1	1	1	0	1	1	1	1	0	1	1	1	1	0	1	1
1	1	1	1	0	1	1	1	0	0	0	0	1	1	1	0	1	1	1	1	0	1	1	1	1	0	1	1
1	1	1	1	0	1	1	1	1	1	0	0	1	0	0	1	1	1	1	1	0	1	0	0	1	0	1	1
1	1	1	1	0	1	1	1	0	1	0	0	1	1	1	0	1	1	1	1	1	1	1	1	1	0	1	1
1	1	1	1	1	1	1	1	1	1	1	1	1	1	1	0	1	1	1	1	1	1	1	1	1	1	1	1
1	1	1	1	1	1	1	1	1	1	1	1	1	1	1	0	1	1	1	1	1	1	1	1	1	1	1	1
1	1	1	1	0	1	1	1	0	1	0	0	1	1	1	0	1	1	1	1	0	1	0	1	1	0	1	1
1	1	1	1	0	1	1	1	0	1	0	0	1	1	1	0	1	1	1	1	1	1	1	1	1	0	1	1
1	1	1	1	0	1	1	1	0	1	0	0	1	0	1	0	1	1	1	1	0	1	0	0	1	0	1	1
1	1	1	1	1	1	1	1	1	1	1	1	1	1	1	0	1	1	1	1	1	1	1	1	1	1	1	1
1	1	1	1	1	1	1	1	1	1	1	1	1	1	1	0	1	1	1	1	1	1	1	1	1	1	1	1
1	1	1	1	0	1	1	1	0	1	0	0	1	1	1	0	1	1	1	1	1	1	1	1	1	0	1	1
1	1	1	1	0	1	1	1	0	1	1	0	1	1	1	0	1	1	1	1	1	1	1	1	1	0	1	1
1	1	1	1	1	1	1	1	1	1	1	1	1	1	1	0	1	1	1	1	1	1	1	1	1	1	1	1
1	1	1	1	1	1	1	1	1	1	1	1	1	1	1	0	1	1	1	1	1	1	1	1	1	1	1	1
1	1	1	1	0	1	1	1	0	1	0	0	1	1	1	0	1	1	1	1	0	1	1	1	1	0	1	1
1	1	1	1	1	1	1	1	1	1	1	1	1	1	1	0	1	1	1	1	1	1	1	1	1	1	1	1
1	1	1	1	0	1	1	1	0	1	0	0	1	1	1	0	1	1	1	1	1	1	1	1	1	0	1	1
1	1	1	1	0	1	1	1	0	1	0	0	1	1	1	0	1	1	1	1	0	1	1	1	1	0	1	1
1	1	1	1	1	1	1	1	1	1	1	1	1	1	1	0	1	1	1	1	1	1	1	1	1	1	1	1
1	1	1	1	0	1	1	1	0	1	1	0	1	1	1	0	1	1	1	1	1	1	1	0	1	0	1	1
1	1	1	1	0	1	1	1	0	1	0	0	1	1	1	0	1	1	1	1	0	1	0	1	1	0	1	1
1	1	1	1	0	1	1	1	0	0	0	0	1	1	1	0	1	1	1	1	0	1	1	1	1	0	1	1
1	1	1	1	0	1	1	1	0	1	0	0	1	1	1	0	1	1	1	1	1	1	1	1	1	0	1	1
1	1	1	1	0	1	1	1	0	1	0	0	1	1	1	0	1	1	1	1	0	1	1	1	1	0	1	1
1	1	1	1	0	1	1	1	0	0	0	0	1	0	1	0	1	1	1	1	0	1	1	0	1	0	1	1
1	1	1	1	0	1	1	1	0	0	0	0	1	0	1	0	1	1	1	1	0	1	1	0	1	0	1	1
1	1	1	1	0	1	1	1	0	1	0	0	1	1	1	0	1	1	1	1	1	1	1	1	1	0	1	1
1	1	1	1	1	1	1	1	1	1	1	1	1	1	1	0	1	1	1	1	1	1	1	1	1	1	1	1
1	1	1	1	0	1	1	1	0	1	0	0	1	1	1	1	1	1	1	1	0	1	1	1	1	0	1	1
1	1	1	1	0	1	1	1	0	0	0	0	1	1	1	0	1	1	1	1	1	1	1	1	1	0	1	1
1	1	1	1	1	1	1	1	1	1	1	1	1	1	1	0	1	1	1	1	1	1	1	1	1	1	1	1
1	1	1	1	1	1	1	1	1	1	0	0	1	1	1	0	1	1	1	1	1	1	1	1	1	0	1	1
1	1	1	1	1	1	1	1	1	1	0	0	1	1	1	0	1	1	1	1	1	1	1	1	1	0	1	1
1	1	1	1	1	1	1	1	1	1	1	1	1	1	1	0	1	1	1	1	1	1	1	1	1	1	1	1
1	1	1	1	0	1	1	1	0	1	0	0	1	1	1	1	1	1	1	1	0	1	1	1	1	0	1	1
1	1	1	1	0	1	1	1	0	1	0	0	1	1	1	0	1	1	1	1	0	1	1	1	1	0	1	1
1	1	1	1	0	1	1	1	0	0	0	0	1	0	1	0	1	1	1	1	0	1	0	1	1	0	1	1
1	1	1	1	1	1	1	1	1	1	1	1	1	1	1	0	1	1	1	1	1	1	1	1	1	1	1	1

1	1	1	1	0	1	1	1	0	1	0	0	1	1	1	0	1	1	1	1	1	1	1	1	1	0	1	1
1	1	1	1	0	1	1	1	0	0	0	0	1	1	1	0	1	1	1	1	0	1	1	1	1	0	1	1
1	1	1	1	0	1	1	1	1	1	1	0	1	1	1	1	1	1	1	1	1	1	1	1	1	0	1	1
1	1	1	1	0	1	1	1	0	1	0	0	1	1	1	0	1	1	1	1	1	1	1	1	1	0	1	1
1	1	1	1	0	1	1	1	0	1	0	0	1	0	1	0	1	1	1	1	0	1	1	0	1	0	1	1
1	1	1	1	1	1	1	1	1	1	1	1	1	1	1	0	1	1	1	1	1	1	1	1	1	1	1	1
1	1	1	1	0	1	1	1	0	1	0	0	1	1	1	0	1	1	1	1	1	1	1	1	1	0	1	1
1	1	1	1	0	1	1	1	0	0	0	0	1	1	1	0	1	1	1	1	0	1	1	1	1	0	1	1
1	1	1	1	0	1	1	1	0	0	0	0	1	1	1	0	1	1	1	1	1	1	1	1	1	0	1	1
1	1	1	1	0	1	1	1	0	0	0	0	1	1	1	0	1	1	1	1	0	1	1	1	1	0	1	1
1	1	1	1	0	1	1	1	0	1	0	0	1	1	1	0	1	1	1	1	1	1	1	1	1	0	1	1
1	1	1	1	0	1	1	1	0	0	0	0	1	1	1	0	1	1	1	1	0	1	1	1	1	0	1	1
1	1	1	1	0	1	1	1	0	1	0	0	1	1	1	0	1	1	1	1	0	1	1	1	1	0	1	1
1	1	1	1	0	1	1	1	0	1	0	0	1	1	1	0	1	1	1	1	0	1	1	1	1	0	1	1
1	1	1	1	0	1	1	1	0	1	0	0	1	0	1	0	1	1	1	1	0	1	1	0	1	0	1	1
1	1	1	1	0	1	1	1	0	1	0	0	1	1	1	1	1	1	1	1	0	1	1	1	1	0	1	1
1	1	1	1	0	1	1	1	0	0	0	0	1	1	1	0	1	1	1	1	0	1	1	1	1	0	1	1
1	1	1	1	0	1	1	1	0	0	0	0	1	1	1	0	1	1	1	1	1	1	1	1	1	0	1	1
1	1	1	1	0	1	1	1	0	1	0	0	1	0	1	0	1	1	1	1	1	1	0	0	1	0	1	1
1	1	1	1	0	1	1	1	0	0	0	0	1	0	0	1	1	1	1	1	0	1	1	0	1	0	1	1
1	1	1	1	1	1	1	1	1	1	0	0	1	1	1	0	1	1	1	1	1	1	1	1	1	0	1	1
1	1	1	1	1	1	1	1	1	1	0	0	1	1	1	0	1	1	1	1	1	1	1	1	1	0	1	1
1	1	1	1	0	1	1	1	0	0	0	0	1	1	1	1	1	1	1	1	0	1	1	1	1	0	1	1
1	1	1	1	1	1	1	1	1	1	1	1	1	1	1	0	1	1	1	1	1	1	1	1	1	1	1	1
1	1	1	1	0	1	1	1	0	0	0	0	1	1	1	0	1	1	1	1	1	1	1	1	1	0	1	1
1	1	1	1	0	1	1	1	0	1	0	0	1	0	1	0	1	1	1	1	0	1	1	0	1	0	1	1
1	1	1	1	0	1	1	1	1	1	1	0	1	1	1	1	1	1	1	1	1	1	1	1	1	0	1	1
1	1	1	1	1	1	1	1	1	1	0	0	1	1	1	0	1	1	1	1	1	1	1	1	1	0	1	1
1	1	1	1	1	1	1	1	1	1	0	0	1	1	1	0	1	1	1	1	1	1	1	1	1	0	1	1
1	1	1	1	1	1	1	1	1	0	1	1	1	1	1	0	1	1	1	1	1	1	1	1	1	1	1	1
1	1	1	1	1	1	1	1	1	0	1	1	1	1	1	0	1	1	1	1	1	1	1	1	1	1	1	1
1	1	1	1	1	1	1	1	1	1	0	0	1	1	1	0	1	1	1	1	1	1	1	1	1	0	1	1
1	1	1	1	0	1	1	1	0	1	0	0	1	0	1	1	1	1	1	1	0	1	0	0	1	0	1	1
1	1	1	1	1	1	1	1	1	1	0	0	1	1	1	0	1	1	1	1	1	1	1	1	1	0	1	1
1	1	1	1	0	1	1	1	0	1	0	0	1	1	1	0	1	1	1	1	0	1	1	1	1	0	1	1
1	1	1	1	1	1	1	1	1	1	1	1	1	1	1	0	1	1	1	1	1	1	1	1	1	1	1	1
1	1	1	1	1	1	1	1	1	1	0	0	1	1	1	0	1	1	1	1	1	1	1	1	1	0	1	1
1	1	1	1	0	1	1	1	0	1	0	0	1	1	1	0	1	1	1	1	0	1	1	1	1	0	1	1
1	1	1	1	1	1	1	1	1	1	1	1	1	1	1	0	1	1	1	1	1	1	1	1	1	1	1	1
1	1	1	1	1	1	1	1	1	1	0	0	1	1	1	0	1	1	1	1	1	1	1	1	1	0	1	1
1	1	1	1	0	1	1	1	0	0	0	0	1	1	1	0	1	1	1	1	1	1	1	1	1	0	1	1
1	1	1	1	0	1	1	1	0	1	0	0	1	1	1	0	1	1	1	1	1	1	1	1	1	0	1	1
1	1	1	1	0	1	1	1	0	1	0	0	1	1	1	0	1	1	1	1	0	1	0	1	1	0	1	1
1	1	1	1	0	1	1	1	0	1	0	0	1	1	1	0	1	1	1	1	0	1	0	1	1	0	1	1
1	1	1	1	0	1	1	1	0	1	0	0	1	1	1	0	1	1	1	1	0	1	1	1	1	0	1	1
1	1	1	1	0	1	1	1	0	1	0	0	1	1	1	0	1	1	1	1	0	1	1	1	1	0	1	1
1	1	1	1	0	1	1	1	0	1	0	0	1	1	1	0	1	1	1	1	0	1	0	1	1	0	1	1
1	1	1	1	0	1	1	1	0	0	0	0	1	1	1	0	1	1	1	1	1	1	1	1	1	0	1	1
1	1	1	1	0	1	1	1	0	1	0	0	1	1	1	0	1	1	1	1	0	1	1	1	1	0	1	1
1	1	1	1	0	1	1	1	0	1	0	0	1	1	1	0	1	1	1	1	0	1	1	1	1	0	1	1
1	1	1	1	1	1	1	1	1	1	1	1	1	1	1	0	1	1	1	1	1	1	1	1	1	1	1	1
1	1	1	1	0	1	1	1	0	0	0	0	1	1	1	0	1	1	1	1	0	1	1	1	1	0	1	1
1	1	1	1	0	1	1	1	0	1	0	0	1	1	1	0	1	1	1	1	0	1	0	1	1	0	1	1

1	1	1	1	0	1	1	1	0	1	0	0	1	0	1	0	1	1	1	1	0	1	1	0	1	0	1	1
1	1	1	1	0	1	1	1	0	1	0	0	1	0	1	1	1	1	1	1	0	1	0	0	1	0	1	1
1	1	1	1	1	1	1	1	1	1	0	0	1	1	1	0	1	1	1	1	1	1	1	1	1	0	1	1
1	1	1	1	1	1	1	1	1	1	1	1	1	1	1	0	1	1	1	1	1	1	1	1	1	1	1	1
1	1	1	1	0	1	1	1	0	1	0	0	1	1	1	0	1	1	1	1	0	1	1	0	1	0	1	1
1	1	1	1	1	1	1	1	1	1	0	0	1	1	1	0	1	1	1	1	1	1	1	1	1	0	1	1
1	1	1	1	0	1	1	1	0	0	0	0	1	1	1	0	1	1	1	1	0	1	1	1	1	0	1	1
1	1	1	1	0	1	1	1	0	0	0	0	1	1	1	0	1	1	1	1	1	1	1	1	1	0	1	1
1	1	1	1	0	1	1	1	0	1	0	0	1	1	1	0	1	1	1	1	0	1	0	1	1	0	1	1
1	1	1	1	0	1	1	1	0	1	1	0	1	1	1	0	1	1	1	1	1	1	1	1	1	0	1	1

cps 2A	dlt A	dpp IV	endo SS	en o	gh 92	htp sA	hyl A	lh k	l h r	ma nN	mn mE	pe pT	pg dA	prs A	rfe A	srt A	ssn A	tr ag	yz pA	Sa lR	Sa lk	na dR	Rgg TR	rto A
1	1	1	1	1	1	1	0	1	1	1	1	1	1	1	0	1	1	1	1	0	0	0	0	1
1	1	1	1	1	1	1	0	1	1	1	1	1	1	1	0	1	1	1	1	0	0	0	0	1
1	1	1	0	1	0	1	0	1	1	1	1	1	1	1	0	1	1	1	1	0	0	0	0	1
1	1	1	0	1	0	1	0	1	1	1	1	1	1	1	0	1	1	1	1	0	0	0	0	1
1	1	1	0	1	0	1	0	1	1	0	1	1	1	1	0	1	1	1	0	0	0	0	1	1
1	1	1	1	1	1	1	1	1	1	1	1	1	1	1	1	1	1	1	1	0	0	1	1	1
1	1	1	0	1	0	1	0	1	1	1	1	1	1	1	0	1	1	0	1	0	0	0	0	1
1	1	1	1	1	1	1	0	0	0	1	1	1	1	1	0	1	1	1	0	1	1	0	0	1
1	0	1	1	1	1	1	0	0	0	1	1	1	1	1	0	1	1	1	0	0	0	0	0	1
1	1	1	0	1	0	1	0	1	1	1	1	1	1	1	0	1	1	1	1	0	0	0	0	1
1	1	1	1	1	1	1	1	1	1	1	1	1	1	1	1	1	1	1	1	0	0	1	1	1
1	1	1	1	1	1	1	0	1	1	1	1	1	1	1	1	1	1	1	1	0	0	1	1	1
1	1	1	0	1	0	1	0	1	1	1	1	1	1	1	0	1	1	0	1	0	0	0	0	1
1	1	1	1	1	1	1	1	1	1	1	1	1	1	1	1	1	1	1	1	0	0	1	1	1
1	1	1	1	1	1	1	1	1	1	1	1	1	1	1	1	1	1	1	1	0	0	1	1	1
1	1	1	1	1	1	1	1	1	1	1	1	1	1	1	1	1	1	1	1	0	0	1	1	1
1	1	1	1	1	1	1	0	1	1	0	1	1	1	1	0	1	1	0	1	0	0	0	0	1
1	1	1	1	1	1	1	1	1	1	1	1	1	1	1	1	1	1	1	1	0	0	1	1	1
1	1	1	0	1	0	1	0	1	1	1	1	1	1	1	0	1	1	0	1	0	0	0	0	1
1	1	1	1	1	1	1	1	1	1	1	1	1	1	1	1	1	1	1	1	0	0	1	1	1
1	1	1	0	1	0	1	0	1	1	1	1	1	1	1	0	1	1	1	1	0	0	0	0	1
1	1	1	1	1	1	1	0	1	1	1	1	1	1	1	0	1	1	0	1	0	0	0	1	1
1	1	1	0	1	0	1	0	1	1	1	1	1	1	1	0	1	1	0	1	0	0	0	0	1
1	1	1	0	1	0	1	0	1	1	1	1	1	1	1	0	1	1	1	1	0	0	0	0	1
1	1	1	1	1	1	1	1	1	1	1	1	1	1	1	1	1	1	1	1	0	0	1	1	1
1	1	1	0	1	0	1	0	1	1	1	1	1	1	1	0	1	1	1	1	0	0	0	0	1
1	1	1	0	1	0	1	0	1	1	1	1	1	1	1	0	1	0	1	0	0	0	0	0	1
1	1	1	1	1	1	1	0	1	1	1	1	1	1	1	0	1	1	1	1	0	0	0	0	1
1	1	1	1	1	1	1	0	1	1	1	1	1	1	1	0	1	1	1	1	0	0	0	0	1
1	1	1	0	1	0	1	0	1	1	1	1	1	1	1	0	1	1	1	1	0	0	0	0	1
1	1	1	1	1	1	1	0	1	1	0	1	1	1	1	0	1	1	0	1	0	0	0	0	1
1	1	1	1	1	1	1	1	1	1	1	1	1	1	1	1	1	1	1	1	0	0	1	1	1
1	1	1	1	1	1	1	1	1	1	1	1	1	1	1	1	1	1	1	1	0	0	1	1	1
1	1	1	1	1	1	1	1	1	1	1	1	1	1	1	1	1	1	1	1	0	0	1	1	1
1	1	1	0	1	0	1	0	1	1	1	1	1	1	1	0	1	1	1	1	0	0	0	0	1
1	1	1	1	1	1	1	1	1	1	1	1	1	1	1	1	1	1	1	1	0	0	1	1	1
1	1	1	0	1	0	1	0	1	1	1	1	1	1	1	0	1	1	1	1	0	0	0	0	1
1	1	1	0	1	0	1	0	1	1	1	1	1	1	1	0	1	1	1	1	0	0	0	0	1
1	1	1	1	1	1	1	0	1	1	1	1	1	1	1	0	1	1	1	1	0	0	0	0	1
1	1	1	1	1	1	1	0	1	1	1	1	1	1	1	0	1	1	1	1	0	0	0	0	1
1	1	1	1	1	1	1	0	1	1	1	1	1	1	1	0	1	1	1	1	0	0	0	0	1

1	1	1	1	1	1	1	1	1	1	1	1	1	1	1	1	1	1	1	1	0	0	1	1	1
1	1	1	1	1	1	1	1	1	1	1	1	1	1	1	1	1	1	1	1	0	0	1	1	1
1	1	1	1	1	1	1	1	1	1	1	1	1	1	1	1	1	1	1	1	0	0	1	1	1
1	1	1	1	1	1	1	1	1	1	1	1	1	1	1	1	1	1	1	1	0	0	1	1	1
1	1	1	0	1	0	1	0	1	1	1	1	1	1	1	0	1	1	1	1	0	0	0	0	1
1	1	1	1	1	1	1	1	1	1	1	1	1	1	1	1	1	1	1	1	0	0	1	1	1
1	1	1	0	1	0	1	0	1	1	1	1	1	1	1	0	1	1	1	1	0	0	0	0	1
1	1	1	1	1	1	1	0	1	1	1	1	1	1	1	0	1	1	1	1	0	0	0	0	1
1	1	1	0	1	0	1	0	1	1	1	1	1	1	1	0	1	1	1	1	0	0	0	0	1
1	1	1	1	1	1	1	1	1	1	1	1	1	1	1	1	1	1	1	1	0	0	1	1	1
1	1	1	0	1	0	1	0	1	1	1	1	1	1	1	0	1	1	1	1	0	0	0	0	1
1	1	1	1	1	1	1	0	1	1	1	1	1	1	1	0	1	1	1	1	0	0	0	0	1
1	1	1	0	1	0	1	0	1	1	1	1	1	1	1	0	1	1	1	1	0	0	0	0	1
1	1	1	1	1	1	1	0	1	1	1	1	1	1	1	1	1	1	0	1	0	0	1	1	1
1	1	1	0	1	0	1	0	1	1	1	1	1	1	1	0	1	1	0	1	0	0	0	0	1
1	1	1	0	1	0	1	0	1	1	1	1	1	1	1	0	1	1	1	1	0	0	0	0	1
1	1	1	1	1	1	1	1	1	1	1	1	1	1	1	1	1	1	1	1	0	0	1	1	1
1	1	1	1	1	1	1	1	1	1	1	1	1	1	1	1	1	1	1	1	0	0	1	1	1
1	1	1	1	1	1	1	1	1	1	1	1	1	1	1	1	1	1	1	1	0	0	1	1	1
1	1	1	1	1	1	1	0	1	1	1	1	1	1	1	0	1	1	1	1	0	0	0	0	1
1	1	1	1	1	1	1	0	1	1	0	1	1	1	1	0	1	1	0	1	0	0	0	0	1
1	1	1	1	1	1	1	0	1	1	1	1	1	1	1	0	1	1	1	1	0	0	0	1	1
1	1	1	0	1	0	1	0	1	1	1	1	1	1	1	0	1	1	1	1	0	0	0	0	1
1	1	1	1	1	1	1	1	1	1	1	1	1	1	1	1	1	1	1	1	0	0	1	1	1
1	1	1	1	1	1	1	1	1	1	1	1	1	1	1	1	1	1	1	1	0	0	1	1	1
1	1	1	1	1	1	1	0	1	1	0	1	1	1	1	0	1	1	0	1	0	0	0	0	1
1	1	1	0	1	0	1	0	1	1	1	1	1	1	1	0	1	1	1	1	0	0	0	0	1
1	0	1	1	1	1	1	0	0	0	1	1	1	1	1	0	1	1	0	0	0	0	0	0	1
1	1	1	1	1	1	1	1	1	1	1	1	1	1	1	1	1	1	1	1	0	0	1	1	1
1	1	1	1	1	1	1	1	1	1	1	1	1	1	1	1	1	1	1	1	0	0	1	1	1
1	1	1	0	1	0	1	0	1	1	1	1	1	1	1	0	1	1	0	1	0	0	0	0	1
1	1	1	1	1	1	1	1	1	1	1	1	1	1	1	1	1	1	1	1	0	0	1	1	1
1	1	1	0	1	0	1	0	1	1	1	1	1	1	1	0	1	1	1	1	0	0	0	0	1
1	1	1	1	1	1	1	1	1	1	1	1	1	1	1	1	1	1	1	1	0	0	1	1	1
1	1	1	0	1	0	1	0	1	1	1	1	1	1	1	0	1	1	0	1	0	0	0	0	1
1	1	1	0	1	0	1	0	1	1	1	1	1	1	1	0	1	1	1	1	0	0	0	0	1
1	1	1	1	1	1	1	0	1	1	0	1	1	1	1	0	1	0	1	0	0	0	0	1	1
1	1	1	1	1	1	1	0	1	1	1	1	1	1	1	0	1	1	1	1	0	0	0	0	1
1	1	1	1	1	1	1	1	1	1	1	1	1	1	1	1	1	1	1	1	0	0	1	1	1
1	1	1	1	1	1	1	1	1	1	1	1	1	1	1	1	1	1	1	1	0	0	1	1	1
1	1	1	1	1	1	1	0	1	1	1	1	1	1	1	0	1	1	1	1	0	0	0	0	1
1	1	1	1	1	1	1	0	1	1	1	1	1	1	1	0	1	1	1	1	0	0	0	0	1
1	1	1	1	1	1	1	0	1	1	1	1	1	1	1	0	1	1	1	1	0	0	0	0	1
1	1	1	1	1	1	1	1	1	1	1	1	1	1	1	1	1	1	1	1	0	0	1	1	1
1	1	1	1	1	1	1	1	1	1	1	1	1	1	1	1	1	1	1	1	0	0	1	1	1
1	1	1	1	1	1	1	0	1	1	1	1	1	1	1	0	1	1	1	1	0	0	0	0	1
1	1	1	1	1	1	1	0	1	1	1	1	1	1	1	0	1	1	1	1	0	0	0	0	1
1	1	1	1	1	1	1	1	1	1	1	1	1	1	1	1	1	1	1	1	0	0	1	1	1
1	1	1	1	1	1	1	1	1	1	1	1	1	1	1	1	1	1	1	1	0	0	1	1	1
1	1	1	0	1	0	1	0	1	1	1	1	1	1	1	0	1	1	1	1	0	0	0	0	1
1	1	1	1	1	1	1	1	1	1	1	1	1	1	1	1	1	1	1	1	0	0	1	1	1
1	1	1	1	1	1	1	0	1	1	1	1	1	1	1	0	1	1	1	1	0	0	0	0	1
1	1	1	0	1	0	1	0	1	1	1	1	1	1	1	0	1	1	1	1	0	0	0	0	1

1	1	1	1	1	1	1	1	1	1	1	1	1	1	1	1	1	1	1	1	0	0	1	1	1
1	1	1	0	1	0	1	0	1	1	1	1	1	1	1	0	1	1	1	1	1	1	0	0	1
1	1	1	1	1	1	1	0	1	1	1	1	1	1	1	0	1	1	1	1	0	0	0	0	1
1	1	1	0	1	0	1	0	1	1	1	1	1	1	1	0	1	1	1	1	0	0	0	0	1
1	1	1	1	1	1	1	0	1	1	1	1	1	1	1	0	1	1	1	1	0	0	0	0	1
1	1	1	0	1	0	1	0	1	1	1	1	1	1	1	0	1	1	1	1	0	0	0	0	1
1	1	1	1	1	1	1	0	1	1	1	1	1	1	1	0	1	1	1	1	0	0	0	0	1
1	1	1	1	1	1	1	0	1	1	1	1	1	1	1	0	1	1	1	1	0	0	0	0	1
1	1	1	1	1	1	1	0	1	1	1	1	1	1	1	0	1	1	1	1	0	0	0	0	1
1	1	1	1	1	1	1	1	1	1	1	1	1	1	1	1	1	1	1	1	0	0	1	1	1
1	1	1	1	1	1	1	0	1	1	1	1	1	1	1	0	1	1	1	1	0	0	0	0	1
1	1	1	1	1	1	1	0	1	1	1	1	1	1	1	0	1	1	1	1	0	0	0	0	1
1	1	1	1	1	1	1	1	1	1	1	1	1	1	1	1	1	1	1	1	0	0	1	1	1
1	1	1	1	1	1	1	1	1	1	1	1	1	1	1	1	1	1	1	1	0	0	1	1	1
1	1	1	1	1	1	1	1	1	1	1	1	1	1	1	1	1	1	1	1	0	0	1	1	1
1	1	1	1	1	1	1	1	1	1	1	1	1	1	1	1	1	1	1	1	0	0	1	1	1
1	1	1	0	1	0	1	0	1	1	1	1	1	1	1	0	1	1	1	1	0	0	0	0	1
1	1	1	0	1	0	1	0	1	1	1	1	1	1	1	0	1	1	1	1	0	0	0	0	1
1	1	1	1	1	1	1	0	1	1	1	1	1	1	1	0	1	1	0	1	0	0	0	0	1
1	1	1	1	1	1	1	1	1	1	1	1	1	1	1	1	1	1	1	1	0	0	0	0	1
1	1	1	1	1	1	1	0	1	1	1	1	1	1	1	0	1	1	1	1	0	0	0	0	1
1	1	1	0	1	0	1	0	1	1	1	1	1	1	1	0	1	1	0	1	0	0	0	1	1
1	1	1	1	1	1	1	0	1	1	1	1	1	1	1	0	1	1	1	1	0	0	0	0	1
1	1	1	1	1	1	1	0	1	1	1	1	1	1	1	0	1	1	1	1	0	0	0	0	1
1	0	1	1	1	1	1	0	0	0	1	1	1	1	1	0	1	1	1	0	0	0	1	1	1
1	1	1	1	1	1	1	1	1	1	1	1	1	1	1	1	1	1	1	1	0	0	0	0	1
1	1	1	1	1	1	1	0	1	1	1	1	1	1	1	0	1	1	1	1	0	0	0	0	1
1	1	1	0	1	0	1	0	1	1	1	1	1	1	1	0	1	1	0	1	0	0	0	0	1
1	1	1	1	1	1	1	0	1	1	0	1	1	1	1	0	1	1	0	1	0	0	0	0	1
1	1	1	0	1	0	1	0	1	1	1	1	1	1	1	0	1	1	0	1	0	0	0	0	1
1	1	1	1	1	1	1	0	1	1	1	1	1	1	1	0	1	1	1	1	0	0	0	0	1
1	1	1	0	1	0	1	0	1	1	1	1	1	1	1	0	1	1	1	1	0	0	0	0	1
1	1	1	0	1	0	1	0	1	1	1	1	1	1	1	0	1	1	1	1	0	0	0	0	1
1	1	1	0	1	0	1	0	1	1	1	1	1	1	1	0	1	1	1	1	0	0	0	0	1
1	0	1	1	1	1	1	0	0	0	1	1	1	1	1	0	1	1	1	0	0	0	0	0	1
1	1	1	1	1	1	1	0	1	1	1	1	1	1	1	0	1	1	1	1	0	0	0	0	1
1	1	1	0	1	0	1	0	1	1	1	1	1	1	1	0	1	1	1	1	0	0	0	0	1
1	1	1	1	1	1	1	0	1	1	0	1	1	1	1	0	1	1	0	1	1	1	0	0	1
1	1	1	1	1	1	1	0	1	1	1	1	1	1	1	0	1	1	1	1	0	0	0	0	1
1	1	1	1	1	1	1	0	0	0	1	1	1	1	1	0	1	1	0	0	0	0	1	1	1
1	1	1	1	1	1	1	1	1	1	1	1	1	1	1	1	1	1	1	1	0	0	1	1	1
1	1	1	1	1	1	1	1	1	1	1	1	1	1	1	1	1	1	1	1	0	0	0	0	1
1	1	1	1	1	1	1	0	1	1	1	1	1	1	1	0	1	1	1	1	0	0	1	1	1
1	1	1	1	1	1	1	1	1	1	1	1	1	1	1	1	1	1	1	1	0	0	0	0	1
1	1	1	1	1	1	1	0	1	1	1	1	1	1	1	0	1	1	1	1	0	0	0	0	1
1	0	1	1	1	1	1	0	0	0	1	1	1	1	1	0	1	1	1	0	1	0	0	1	1
1	1	1	1	1	1	1	0	1	1	1	1	1	1	1	0	1	1	1	1	0	0	1	1	1
1	1	1	1	1	1	1	1	1	1	1	1	1	1	1	1	1	1	1	1	0	0	1	1	1
1	1	1	1	1	1	1	1	1	1	1	1	1	1	1	1	1	1	1	1	0	0	1	1	1
1	1	1	1	1	1	1	0	1	1	1	1	1	1	1	1	1	1	1	1	0	0	1	1	1
1	1	1	1	1	1	1	0	1	1	1	1	1	1	1	1	1	1	1	1	0	0	1	1	1
1	1	1	1	1	1	1	1	1	1	1	1	1	1	1	1	1	1	1	1	0	0	0	0	1
1	0	1	0	1	0	1	0	1	1	0	1	1	1	1	0	1	1	1	0	0	0	1	1	1

1	1	1	1	1	1	1	1	1	1	1	1	1	1	1	1	1	1	1	1	0	0	0	0	1
1	1	1	0	1	0	1	0	1	1	1	1	1	1	1	0	1	1	1	1	0	0	1	1	1
1	1	1	1	1	1	1	1	1	1	1	1	1	1	1	1	1	1	1	1	0	0	1	1	1
1	1	1	1	1	1	1	1	1	1	1	1	1	1	1	1	1	1	1	1	0	0	0	0	1
1	1	1	0	1	0	1	0	1	1	1	1	1	1	1	0	1	1	1	1	0	0	1	1	1
1	1	1	1	1	1	1	1	1	1	1	1	1	1	1	1	1	1	1	1	0	0	1	1	1
1	1	1	1	1	1	1	1	1	1	1	1	1	1	1	1	1	1	1	1	0	0	0	0	1
1	1	1	1	1	1	1	0	1	1	1	1	1	1	1	0	1	1	1	1	0	0	0	0	1
1	1	1	1	1	1	1	0	1	1	1	1	1	1	1	0	1	1	1	1	0	0	0	0	1
1	1	1	1	1	1	1	0	1	1	1	1	1	1	1	0	1	1	1	1	0	0	0	0	1
1	1	1	1	1	1	1	0	1	1	1	1	1	1	1	0	1	1	1	1	0	0	0	0	1
1	1	1	0	1	0	1	0	1	1	1	1	1	1	1	0	1	1	1	1	0	0	0	0	1
1	1	1	0	1	0	1	0	1	1	1	1	1	1	1	0	1	1	1	1	0	0	0	0	1
1	1	1	1	1	1	1	0	1	1	1	1	1	1	1	0	1	1	1	1	0	0	0	0	1
1	1	1	1	1	1	1	0	1	1	0	1	1	1	1	0	1	1	0	1	0	0	0	0	1
1	1	1	0	1	0	1	0	1	1	1	1	1	1	1	0	1	1	1	1	0	0	0	0	1
1	1	1	0	1	0	1	0	1	1	1	1	1	1	1	0	1	1	1	1	0	0	1	1	1
1	1	1	1	1	1	1	1	1	1	1	1	1	1	1	1	1	1	1	1	0	0	0	0	1
1	1	1	0	1	0	1	0	1	1	1	1	1	1	1	0	1	1	1	1	0	0	0	0	1
1	1	1	1	1	1	1	0	1	1	1	1	1	1	1	0	1	1	1	1	0	0	0	0	1
1	0	1	1	1	1	1	0	0	0	1	1	1	1	1	0	1	1	1	0	0	0	0	0	1
1	0	1	0	1	0	1	0	1	1	0	1	1	1	1	0	1	1	1	0	0	0	1	1	1
1	1	1	1	1	1	1	1	1	1	1	1	1	1	1	1	1	1	1	1	0	0	1	1	1
1	1	1	1	1	1	1	1	1	1	1	1	1	1	1	1	1	1	1	1	0	0	0	0	1
1	1	1	0	1	0	1	0	1	1	1	1	1	1	1	0	1	1	1	0	0	0	1	1	1
1	1	1	1	1	1	1	1	1	1	1	1	1	1	1	1	1	1	1	1	0	0	0	0	1
1	1	1	0	1	0	1	0	1	1	1	1	1	1	1	0	1	1	1	1	0	0	0	0	1
1	1	1	1	1	1	1	0	1	1	0	1	1	1	1	0	1	1	0	1	0	0	0	0	1
1	1	1	1	1	1	1	0	1	1	1	1	1	1	1	0	1	1	1	1	0	0	0	0	1
1	1	1	1	1	1	1	0	1	1	1	1	1	1	1	0	1	1	1	1	0	0	0	0	1

Table S4. The matrix of the presence or absence of ARGs in 177 suis genomes

Table S4. The matrix of the presence or absence of ARGs in 177 *suis* genomes

Strain name	AAC (6')-Ie-APH(2'')-Ia	aad (6)	ANT (9)-Ia	ANT (6)-Ia	APH(3')-IIIa	SAT-4	catA8	cat-TC	fexA	lnuB	lnuC	lsaE	ErmA	ErmB	mel	patA	patB	tet(O)	tet(32)	tet(40)	tet(M)	tet(W)	tet(O/W/32/O)	tet(L)	dfrF	dfrG
C9523	0	0	0	0	0	0	0	0	0	0	0	0	0	1	0	1	1	1	0	0	0	0	0	0	0	0
C9726	0	0	0	0	0	0	0	0	0	0	0	0	0	1	0	1	1	1	0	0	0	0	0	0	0	0
E1120	0	0	0	0	0	0	0	0	0	0	0	0	0	1	0	0	1	1	0	0	0	0	0	0	0	0
E1210	0	0	0	0	0	0	0	0	0	0	0	0	0	1	0	0	1	1	0	0	0	0	0	0	0	0
E1225	0	0	0	0	0	0	0	0	0	0	0	0	0	1	0	1	1	1	0	0	0	0	0	0	0	0
E1529	0	1	0	0	1	1	0	0	0	0	0	0	0	1	0	1	1	1	0	1	0	0	0	0	0	0
E1819	0	0	0	1	0	0	0	0	0	1	0	1	0	1	1	1	1	1	0	0	0	0	0	0	0	0
E1974	0	0	0	0	0	0	0	0	0	0	0	0	0	1	0	1	1	0	0	0	1	0	0	0	0	0
E1991	0	0	0	0	0	0	0	0	0	0	0	0	0	1	0	1	1	1	0	0	0	0	0	0	0	0
E2126	0	0	0	0	0	0	0	0	0	0	0	0	0	1	0	1	1	1	0	0	0	0	0	0	0	0
E2128	0	0	0	1	0	0	0	0	0	0	0	0	0	1	0	1	1	1	0	1	0	0	0	0	0	1
E2309	1	0	0	0	1	1	1	0	0	1	0	1	0	1	0	1	1	1	0	0	0	0	0	0	0	0
E2376	0	0	0	1	0	0	0	0	0	1	0	1	0	0	0	1	1	1	0	0	0	0	0	0	0	0
E2498	0	0	0	1	0	0	0	0	0	1	0	1	0	1	1	1	1	0	0	1	0	0	0	0	0	0

E2592	0	1	0	0	1	1	0	0	0	0	0	0	0	1	0	1	1	1	0	1	0	0	0	0	0	0
E2690	0	1	0	0	1	1	0	0	0	0	0	0	0	1	0	1	1	1	0	1	0	0	0	0	0	0
E2724	0	0	0	0	0	0	0	0	0	0	0	0	0	1	0	1	1	1	0	0	0	0	0	0	0	0
E2741	0	1	0	0	1	1	0	0	0	0	0	0	0	1	0	1	1	1	0	1	0	0	0	0	0	0
E2758	0	0	0	1	0	0	0	0	0	1	0	1	0	1	1	1	1	1	0	0	0	0	0	0	0	0
E2871	0	0	0	0	0	0	0	0	0	0	0	0	0	1	0	1	1	1	0	0	0	0	0	0	0	0
E3038	0	0	0	0	0	0	0	0	0	0	0	0	0	1	0	0	1	1	0	0	0	0	0	0	0	0
E3111	0	0	0	0	0	0	0	0	0	0	0	0	1	1	0	1	1	1	0	1	0	0	0	0	0	0
E3123	0	0	0	0	0	0	0	0	0	0	0	0	0	1	0	1	1	1	0	0	0	0	0	0	0	0
E3145	0	0	0	0	0	0	0	0	0	0	0	0	0	0	0	1	1	0	0	0	0	0	0	0	0	0
E3342	0	1	0	0	1	1	0	0	0	0	0	0	0	1	0	1	1	1	0	1	0	0	0	0	0	0
E3606	0	0	0	0	0	0	0	0	0	0	0	0	1	1	0	1	1	1	0	0	0	0	0	0	0	0
E3937	1	0	0	0	1	1	0	0	0	0	0	0	1	0	0	1	1	0	0	0	0	0	1	0	0	0
E3946	0	0	0	0	0	0	0	0	0	0	1	0	0	1	0	1	1	0	1	0	0	1	0	0	1	0
E3948	0	0	0	0	0	0	0	0	0	0	0	0	0	1	0	1	1	1	0	0	0	0	0	0	0	0
E4081	0	0	0	0	0	0	0	0	0	0	0	0	0	1	0	1	1	1	0	0	0	0	0	0	0	0
E4299	0	0	0	0	0	0	0	0	0	0	0	0	0	1	0	1	1	1	0	0	0	0	0	0	0	0
E4308	0	1	0	0	1	1	0	0	0	0	0	0	0	1	0	1	1	1	0	1	0	0	0	0	0	0
E4343	0	1	0	0	1	1	0	0	0	0	0	0	0	1	0	1	1	1	0	1	0	0	0	0	0	0
E4344	0	1	0	0	1	1	0	0	0	0	0	0	0	1	0	1	1	1	0	1	0	0	0	0	0	0
E4504	0	0	0	0	0	0	0	0	0	0	0	0	0	1	0	1	1	1	0	0	0	0	0	0	0	0
E4513	0	1	0	0	1	1	0	0	0	0	0	0	0	1	0	1	1	1	0	1	0	0	0	0	0	0
E4555	0	0	0	0	0	0	0	0	0	0	0	0	0	1	0	1	1	1	0	0	0	0	0	0	0	0
E4558	0	0	0	0	0	0	0	0	0	0	0	0	0	1	0	1	1	1	0	0	0	0	0	0	0	0
E4843	0	0	0	0	0	0	0	0	0	0	0	0	0	1	0	1	1	1	0	0	0	0	0	0	0	0
E4968	0	0	0	0	0	0	0	0	0	0	1	0	0	1	0	1	1	0	1	0	0	1	0	0	1	0
E5246	0	0	0	0	0	0	0	0	0	0	0	0	0	1	0	1	1	1	0	0	0	0	0	0	0	1
E5271	0	1	0	0	1	1	0	0	0	0	0	0	0	1	0	1	1	1	0	1	0	0	0	0	0	0
E5333	0	1	0	0	1	1	0	0	0	0	0	0	0	1	0	1	1	1	0	1	0	0	0	0	0	0
E5369	0	1	0	0	1	1	0	0	0	0	0	0	0	1	0	1	1	1	0	1	0	0	0	0	0	0
E5450	0	0	0	1	0	0	0	0	0	1	0	1	0	1	1	1	1	0	0	1	0	0	0	0	0	0
E5472	0	0	0	0	0	0	0	0	0	0	0	0	0	1	0	0	1	1	0	0	0	0	0	0	0	0
E5476	0	1	0	0	1	1	0	0	0	0	0	0	0	1	0	1	1	1	0	1	0	0	0	0	0	0
E5606	0	0	0	0	0	0	0	0	0	0	0	0	0	1	0	0	1	1	0	0	0	0	0	0	0	0
E5611	0	0	0	0	0	0	0	0	0	0	0	0	0	1	0	1	1	1	1	0	0	0	0	0	1	0
E5879	0	0	0	0	0	0	0	0	0	0	0	0	1	1	0	1	1	1	0	0	0	0	0	0	0	0
E6114	0	1	0	0	1	1	0	0	0	0	0	0	0	1	0	1	1	1	0	1	0	0	0	0	0	0
E6124	0	0	0	0	0	0	0	0	0	0	0	0	0	1	0	1	1	1	0	0	0	0	0	0	0	0
E6199	0	0	0	0	0	0	0	0	0	0	0	0	0	1	0	1	1	1	0	0	0	0	0	0	0	0
E6207	0	0	0	0	0	0	0	0	0	0	0	0	0	1	0	0	1	1	0	0	0	0	0	0	0	0
E6352	0	0	0	0	0	0	0	0	0	0	0	0	0	0	0	1	1	1	0	1	0	0	0	0	0	0
E6439	0	0	0	0	0	0	0	0	0	0	0	0	0	1	0	1	1	1	0	0	0	0	0	0	0	0
E6444	0	0	0	0	0	0	0	0	0	0	0	0	0	1	0	1	1	1	0	0	0	0	0	0	0	0
E6452	0	1	0	0	1	1	0	0	0	0	0	0	0	1	0	1	1	1	0	1	0	0	0	0	0	0
E6488	0	1	0	0	1	1	0	0	0	0	0	0	0	1	0	1	1	1	0	1	0	0	0	0	0	0
E6504	0	1	0	0	1	1	0	0	0	0	0	0	0	1	0	1	1	1	0	1	0	0	0	0	0	0
E6514	0	0	0	0	0	0	0	0	0	0	0	0	0	1	0	1	1	1	0	0	0	0	0	0	0	0
E6605	0	0	0	0	0	0	0	0	0	0	0	0	0	1	0	1	1	1	0	0	0	0	0	0	0	0

E6640	1	0	0	1	0	0	0	0	0	1	0	1	0	1	0	1	1	1	0	0	0	0	0	0	0	0
E6702	0	0	0	0	0	0	0	0	0	0	0	0	0	1	0	1	1	1	0	0	0	0	0	0	0	1
E6718	0	0	0	1	0	0	0	0	0	1	0	1	0	1	1	1	1	0	0	1	0	0	0	0	0	0
E6886	0	1	0	0	1	1	0	0	0	0	0	0	0	1	0	1	1	1	0	1	0	0	0	0	0	0
E6897	0	0	0	0	0	0	0	0	0	0	0	0	0	1	0	1	1	1	0	0	0	0	0	0	0	0
E7334	0	0	0	0	0	0	0	0	0	0	0	0	0	1	0	1	1	1	0	0	0	0	0	0	0	0
E7403	0	0	0	1	0	0	0	0	0	0	0	0	0	1	0	1	1	0	0	0	1	0	0	0	0	0
E7526	0	1	0	0	1	1	0	0	0	0	0	0	0	1	0	1	1	1	0	1	0	0	0	0	0	0
E7567	0	1	0	0	1	1	0	0	0	0	0	0	0	1	0	1	1	1	0	1	0	0	0	0	0	0
E7616	0	0	0	0	0	0	0	0	0	0	0	0	0	1	0	1	1	1	0	0	0	0	0	0	0	0
E7634	0	1	0	0	1	1	0	0	0	0	0	0	0	1	0	1	1	1	0	1	0	0	0	0	0	0
E7657	0	0	0	0	0	0	0	0	0	0	0	0	0	1	0	1	1	1	0	0	0	0	0	0	0	0
E7695	0	1	0	0	1	1	0	0	0	0	0	0	0	1	0	1	1	1	0	1	0	0	0	0	0	0
E7716	0	0	0	0	0	0	0	0	0	0	0	0	0	1	0	1	1	1	0	0	0	0	0	0	0	0
E7750	0	0	0	0	0	0	0	0	0	0	0	0	0	1	0	0	1	1	0	0	0	0	0	0	0	0
E7885	0	0	0	1	1	0	0	0	0	0	0	0	1	0	0	1	1	0	0	0	0	0	1	0	0	0
E7912	0	0	0	0	0	0	0	0	0	0	0	0	0	1	0	1	1	1	0	0	0	0	0	0	0	0
E8036	0	1	0	0	1	1	0	0	0	0	0	0	0	1	0	1	1	1	0	1	0	0	0	0	0	0
E8168	0	1	0	0	1	1	0	0	0	0	0	0	0	1	0	1	1	1	0	1	0	0	0	0	0	0
E8229	0	0	0	0	0	0	0	0	0	0	0	0	0	1	0	1	1	1	0	0	0	0	0	0	0	0
E8300	0	0	0	0	0	0	0	0	0	0	0	0	0	1	0	1	1	1	0	0	0	0	0	0	0	0
E8420	0	0	0	0	0	0	0	0	1	1	0	1	1	0	0	1	1	0	0	0	0	0	1	0	0	0
E8650	0	1	0	0	1	1	0	0	0	0	0	0	0	1	0	1	1	1	0	1	0	0	0	0	0	0
E8772	0	1	0	0	1	1	0	0	0	0	0	0	0	1	0	1	1	1	0	1	0	0	0	0	0	0
E8888	0	0	0	0	0	0	0	0	0	0	0	0	0	1	0	1	1	1	0	0	0	0	0	0	0	0
E8894	0	0	0	0	0	0	0	0	0	0	0	0	0	1	0	1	1	1	0	0	0	0	0	0	0	0
E8996	0	1	0	0	1	1	0	0	0	0	0	0	0	1	0	1	1	1	0	1	0	0	0	0	0	0
E9142	0	1	0	0	1	1	0	0	0	0	0	0	0	1	0	1	1	1	0	1	0	0	0	0	0	0
E9349	0	0	0	0	0	0	0	0	0	0	0	0	0	1	0	0	1	1	0	0	0	0	0	0	0	0
E9490	0	1	0	0	1	1	0	0	0	0	0	0	0	1	0	1	1	1	0	1	0	0	0	0	0	0
E9696	0	0	0	0	0	0	0	0	0	0	0	0	0	1	0	1	1	1	0	0	0	0	0	0	0	0
E9719	0	0	0	0	0	0	0	0	0	0	0	0	0	1	0	1	1	1	0	0	0	0	0	0	0	0
E9733	0	1	0	0	1	1	0	0	0	0	0	0	0	1	0	1	1	1	0	1	0	0	0	0	0	0
G6942	0	0	0	0	0	0	0	0	0	0	0	0	0	1	0	1	1	1	0	0	0	0	0	0	0	0
J3545	0	0	0	0	0	0	0	0	0	0	0	0	0	1	0	1	1	1	0	0	0	0	0	0	0	0
J5817	0	0	0	0	0	0	0	0	0	0	0	0	0	1	0	0	1	1	0	0	0	0	0	0	0	0
J7160	0	0	0	0	0	0	0	0	0	0	0	0	0	1	0	1	1	1	0	0	0	0	0	0	0	0
J9245	0	0	0	0	0	0	0	0	0	0	0	0	0	1	0	1	1	1	0	0	0	0	0	0	0	0
L2400	0	0	0	1	0	0	0	0	0	1	0	1	0	0	0	1	1	1	0	0	0	0	0	0	0	0
L2401	0	0	0	1	0	0	0	0	0	1	0	1	0	0	0	1	1	1	0	0	0	0	0	0	0	0
L2763	0	0	0	0	0	0	0	0	0	0	0	0	0	1	0	1	1	1	0	0	0	0	0	0	0	0
L2814	0	0	0	1	0	0	0	0	0	1	0	1	0	1	1	1	1	0	0	1	0	0	0	0	0	0
L7545	0	0	0	0	0	0	0	0	0	0	1	0	0	1	0	1	1	0	0	0	0	1	0	0	0	0
M2248	0	0	0	0	0	0	0	0	0	0	0	0	0	1	0	1	1	1	0	0	0	0	0	0	0	0
M6600	0	0	0	1	0	0	0	0	0	0	0	0	0	1	0	1	1	1	0	1	0	0	0	0	0	1
M6937	0	0	0	0	0	0	0	0	0	0	0	0	0	1	0	1	1	1	0	0	0	0	0	0	0	0
M7157	0	0	0	0	0	0	0	0	0	0	0	0	0	1	0	1	1	1	0	0	0	0	0	0	0	0
M9253	0	0	0	1	0	0	0	0	0	0	0	0	0	1	0	1	1	1	0	1	0	0	0	0	0	1

M9403	0	0	0	0	0	0	0	0	0	0	0	0	0	1	0	1	1	1	0	0	0	0	0	0	0	0
N8215	0	0	0	0	0	0	0	0	0	0	0	0	0	1	0	0	1	1	0	0	0	0	0	0	0	0
S6670	1	0	0	1	0	0	0	1	0	0	0	0	0	1	0	1	1	0	0	0	1	0	0	0	0	0
X1706	0	0	0	0	0	0	0	0	0	0	0	0	0	1	0	1	1	1	0	0	0	0	0	0	0	0
X2459	0	0	0	1	0	0	0	0	0	0	0	0	0	1	0	1	1	1	0	0	0	0	0	0	0	0
X3426	0	0	0	0	0	0	0	0	0	0	0	0	0	1	0	1	1	1	0	0	0	0	0	0	0	0
X4751	0	0	0	0	0	0	0	0	0	0	0	0	0	1	0	1	1	1	0	0	0	0	0	0	0	0
X4863	0	0	0	0	0	0	0	0	0	0	0	0	0	1	0	1	1	1	0	0	0	0	0	0	0	0
X5705	0	1	0	0	1	1	0	0	0	0	0	0	0	1	0	1	1	1	0	1	0	0	0	0	0	0
X7400	0	0	0	0	0	0	0	0	0	0	0	0	0	0	0	1	1	1	0	0	0	0	0	0	0	0
X7828	0	0	0	1	0	0	0	0	0	0	0	0	0	1	0	1	1	1	0	0	0	0	0	0	0	0
X9177	0	0	0	0	0	0	0	0	0	0	0	0	0	1	0	1	1	1	0	0	0	0	0	0	0	0
X9285	0	0	0	0	0	0	0	0	0	0	0	0	0	1	0	1	1	1	0	0	0	0	0	0	0	0
X9374	0	0	0	0	0	0	0	0	0	0	0	0	0	1	0	1	1	1	0	0	0	0	0	0	0	0
Y1080	0	0	0	0	0	0	0	0	0	0	0	0	0	1	0	0	1	1	0	0	0	0	0	0	0	0
Y1081	0	0	0	0	0	0	0	0	0	0	0	0	0	1	0	1	1	1	0	0	0	0	0	0	0	1
Y1087	0	0	0	0	0	0	0	0	0	0	0	0	0	1	0	0	1	1	0	0	0	0	0	0	0	0
Y1173	0	0	0	0	0	0	0	0	0	0	0	0	0	1	0	1	1	1	0	0	0	0	0	0	0	0
Y1676	0	0	1	0	0	0	0	0	0	0	0	0	0	0	0	1	1	0	1	0	0	0	0	0	0	0
Y1717	0	0	0	0	0	0	0	0	0	0	0	0	0	1	0	1	1	1	0	0	0	0	0	0	0	0
Y1892	0	0	0	0	0	0	0	0	0	0	0	0	0	1	0	1	1	1	0	0	0	0	0	0	0	0
Y1961	0	0	0	0	0	0	0	0	0	0	0	0	0	1	0	1	1	1	0	0	0	0	0	1	0	0
Y2637	0	0	0	1	0	0	0	0	0	0	0	0	0	1	0	1	1	0	0	0	1	0	0	0	0	0
Y2715	0	0	0	0	0	0	0	0	0	0	0	0	0	1	0	1	1	1	0	0	0	0	0	0	0	0
Y2968	0	0	0	0	0	0	0	0	0	0	0	0	0	1	0	1	1	1	0	0	0	0	0	0	0	0
Y3249	0	0	0	0	0	0	0	0	0	0	1	0	0	1	0	1	1	1	0	0	0	1	0	0	0	0
Y3277	0	1	0	0	1	1	0	0	0	0	0	0	0	1	0	1	1	1	0	0	0	0	0	0	0	0
Y3343	0	0	0	0	0	0	0	0	0	0	0	0	0	1	0	1	1	1	0	0	0	0	0	0	0	0
Y3443	0	0	0	0	0	0	0	0	0	0	0	0	0	1	0	1	1	1	0	0	0	0	0	0	0	0
Y3842	0	0	0	1	0	0	0	0	0	0	0	0	0	1	0	1	1	0	0	0	1	0	0	0	0	0
Y4371	0	0	0	0	0	0	0	0	0	0	0	0	0	1	0	1	1	1	0	0	0	0	0	0	0	0
Y4934	0	0	0	0	0	0	0	0	0	0	0	0	0	1	0	1	1	1	0	0	0	0	0	0	0	0
Y4943	1	0	0	1	1	1	1	0	0	1	0	1	0	0	0	1	1	1	0	0	0	0	0	0	0	1
Y5009	1	0	0	1	1	1	1	0	0	1	0	1	0	0	0	1	1	1	0	0	0	0	0	0	0	0
Y5247	0	0	0	0	0	0	0	0	0	0	0	0	0	1	0	1	1	1	0	0	0	0	0	0	0	0
Y5411	0	0	0	0	0	0	0	0	0	0	0	0	1	1	0	1	1	1	0	0	0	0	0	0	0	0
Y5682	0	0	0	0	0	0	0	0	0	0	0	0	0	1	0	1	1	1	0	0	0	0	0	0	0	0
Y5792	0	0	0	0	0	0	0	0	0	0	0	0	0	1	0	0	1	1	0	0	0	0	0	0	0	0
Y5923	0	0	0	0	0	0	0	0	0	0	0	0	0	1	0	1	1	1	0	0	0	0	0	0	0	0
Y6166	0	0	0	0	0	0	0	0	0	0	0	0	0	1	0	1	1	1	0	0	0	0	0	0	0	0
Y6197	0	0	0	0	0	0	0	0	0	0	0	0	0	1	0	0	1	1	0	0	0	0	0	0	0	0
Y6648	0	0	0	0	0	0	0	0	0	0	0	0	0	1	0	1	1	1	0	0	1	0	0	0	0	0
Y6784	0	0	0	0	0	0	0	0	0	0	0	0	0	1	0	1	1	1	0	0	0	0	0	0	1	0
Y7283	0	0	0	0	0	0	0	0	0	0	0	0	0	1	0	1	1	1	0	0	0	0	0	0	0	0
Y7354	0	0	0	0	0	0	0	0	0	0	0	0	0	1	0	1	1	1	0	0	0	0	0	0	0	0
Y7361	0	0	0	0	0	0	0	0	0	0	0	0	0	1	0	1	1	1	0	0	0	0	0	0	0	0
Y7511	0	0	0	0	0	0	0	0	0	0	0	0	0	1	0	1	1	1	0	0	0	0	0	0	0	0
Y7742	0	0	0	0	0	0	0	0	0	0	0	0	0	1	0	0	1	1	0	0	0	0	0	0	0	0

Y7767	0	0	0	0	0	0	0	0	0	0	0	0	0	1	0	1	1	1	0	0	0	0	0	0	0	1
Y8023	0	0	0	0	0	0	0	0	0	0	0	0	0	1	0	1	1	1	0	0	0	0	0	0	0	0
Y8024	0	0	0	0	0	0	0	0	0	0	0	0	0	1	0	1	1	1	0	0	0	0	0	0	0	0
Y8131	0	0	0	0	0	0	0	0	0	0	0	0	0	1	0	0	1	1	0	0	0	0	0	0	0	0
Y8485	0	0	0	0	0	0	0	0	0	0	0	0	0	1	0	0	1	1	0	0	0	0	0	0	0	0
Y8500	0	1	0	0	1	1	0	0	0	0	0	0	0	1	0	1	1	1	0	1	0	0	0	0	0	0
Y8649	0	0	0	0	0	0	0	0	0	0	0	0	0	1	0	0	1	1	0	0	0	0	0	0	0	0
Y8704	0	0	0	0	0	0	0	0	0	0	0	0	0	1	0	1	1	1	0	0	0	0	0	0	0	0
Y9217	0	0	0	0	0	0	0	0	0	0	0	0	0	1	0	1	1	1	0	0	0	0	0	0	0	0
Y9239	0	0	0	1	0	0	0	0	0	0	0	0	0	0	0	1	1	1	0	0	0	0	0	0	0	0
Y9437	0	0	0	0	0	0	0	0	0	0	0	0	0	1	0	1	1	1	0	0	0	0	0	0	0	0
Y9446	0	1	0	0	1	1	0	0	0	0	0	0	0	1	0	1	1	1	0	1	0	0	0	0	0	0
Y9707	0	0	0	0	1	1	1	0	0	1	0	1	0	1	0	1	1	1	0	0	0	0	0	0	0	0
Y9931	0	0	0	0	0	0	0	0	0	0	0	0	0	1	0	1	1	1	0	0	0	0	0	0	0	0
Z5574	0	0	0	0	0	0	0	0	0	0	0	0	0	1	0	0	1	1	0	0	0	0	0	0	0	0
Z7256	0	0	0	0	0	0	0	0	0	0	0	0	0	1	0	1	1	1	0	0	0	0	0	0	0	0
Z7590	0	0	0	0	0	0	0	0	0	0	0	0	0	1	0	1	1	1	0	0	0	0	0	0	0	0
Z9616	0	0	0	0	0	0	0	0	0	0	0	0	0	1	0	1	1	1	0	0	0	0	0	0	0	0
Z9945	0	0	0	1	0	0	0	0	0	0	0	0	0	0	0	1	1	1	0	0	1	0	0	0	0	0

Table S5. The matrix of the presence or absence of ARGs carried by ICE and IME

Table S5. The matrix of the presence or absence of ARGs carried by ICE and IME																						
Strain name	ARGs carried by ICE																		ARGs carried by IME				
	AAC(6')-Ie-APH(2")-Ia	ANT(6)-Ia	APH(3')-IIIa	ErmA	ErmB	SAT-4	aad(6)	cat-TC	dfrG	lnuB	lsaE	mel	tet(40)	tet(L)	tet(M)	tet(O)	tet(O/W/32/O)	tet(W)	APH(3')-IIIa	ErmA	lnuC	tet(O)	tet(W)
C9523	0	0	0	0	1	0	0	0	0	0	0	0	0	0	0	1	0	0	0	0	0	1	0
C9726	0	0	0	0	1	0	0	0	0	0	0	0	0	0	0	1	0	0	0	0	0	1	0
E1120	0	0	0	0	1	0	0	0	0	0	0	0	0	0	0	1	0	0	0	0	0	1	0
E1210	0	0	0	0	1	0	0	0	0	0	0	0	0	0	0	1	0	0	0	0	0	1	0
E1225	0	0	0	0	1	0	0	0	0	0	0	0	0	0	0	1	0	0	0	0	0	1	0
E1529	0	0	1	0	1	1	1	0	0	0	0	0	1	0	0	1	0	0	1	0	0	1	0
E1819	0	1	0	0	1	0	0	0	0	1	1	1	0	0	0	1	0	0	0	0	0	1	0
E1974	0	0	0	0	1	0	0	0	0	0	0	0	0	0	1	0	0	0	0	0	0	0	0
E1991	0	0	0	0	1	0	0	0	0	0	0	0	0	0	0	1	0	0	0	0	0	1	0
E2126	0	0	0	0	1	0	0	0	0	0	0	0	0	0	0	1	0	0	0	0	0	1	0
E2128	0	1	0	0	1	0	0	0	1	0	0	0	1	0	0	1	0	0	0	0	0	1	0
E2309	1	0	1	0	1	1	0	0	0	1	1	0	0	0	0	1	0	0	1	0	0	1	0
E2376	0	1	0	0	0	0	0	0	0	1	1	0	0	0	0	1	0	0	0	0	0	1	0
E2498	0	1	0	0	1	0	0	0	0	1	1	1	1	0	0	0	0	0	0	0	0	0	0
E2592	0	0	1	0	1	1	1	0	0	0	0	0	1	0	0	1	0	0	1	0	0	1	0
E2690	0	0	1	0	1	1	1	0	0	0	0	0	1	0	0	1	0	0	1	0	0	1	0
E2724	0	0	0	0	1	0	0	0	0	0	0	0	0	0	0	1	0	0	0	0	0	1	0
E2741	0	0	1	0	1	1	1	0	0	0	0	0	1	0	0	1	0	0	1	0	0	1	0
E2758	0	1	0	0	1	0	0	0	0	1	1	1	0	0	0	1	0	0	0	0	0	1	0
E2871	0	0	0	0	1	0	0	0	0	0	0	0	0	0	0	1	0	0	0	0	0	1	0
E3038	0	0	0	0	1	0	0	0	0	0	0	0	0	0	0	1	0	0	0	0	0	1	0
E3111	0	0	0	1	1	0	0	0	0	0	0	0	1	0	0	1	0	0	0	1	0	1	0

E3123	0	0	0	0	1	0	0	0	0	0	0	0	0	0	0	1	0	0	0	0	0	1	0
E3145	0	0	0	0	0	0	0	0	0	0	0	0	0	0	0	0	0	0	0	0	0	0	0
E3342	0	0	1	0	1	1	1	0	0	0	0	0	1	0	0	1	0	0	1	0	0	1	0
E3606	0	0	0	1	1	0	0	0	0	0	0	0	0	0	0	1	0	0	0	1	0	1	0
E3937	1	0	1	1	0	1	0	0	0	0	0	0	0	0	0	0	1	0	1	1	0	0	0
E3946	0	0	0	0	1	0	0	0	0	0	0	0	0	0	0	0	0	1	0	0	1	0	1
E3948	0	0	0	0	1	0	0	0	0	0	0	0	0	0	0	1	0	0	0	0	0	1	0
E4081	0	0	0	0	1	0	0	0	0	0	0	0	0	0	0	1	0	0	0	0	0	1	0
E4299	0	0	0	0	1	0	0	0	0	0	0	0	0	0	0	1	0	0	0	0	0	1	0
E4308	0	0	1	0	1	1	1	0	0	0	0	0	1	0	0	1	0	0	1	0	0	1	0
E4343	0	0	1	0	1	1	1	0	0	0	0	0	1	0	0	1	0	0	1	0	0	1	0
E4344	0	0	1	0	1	1	1	0	0	0	0	0	1	0	0	1	0	0	1	0	0	1	0
E4504	0	0	0	0	1	0	0	0	0	0	0	0	0	0	0	1	0	0	0	0	0	1	0
E4513	0	0	1	0	1	1	1	0	0	0	0	0	1	0	0	1	0	0	1	0	0	1	0
E4555	0	0	0	0	1	0	0	0	0	0	0	0	0	0	0	1	0	0	0	0	0	1	0
E4558	0	0	0	0	1	0	0	0	0	0	0	0	0	0	0	1	0	0	0	0	0	1	0
E4843	0	0	0	0	1	0	0	0	0	0	0	0	0	0	0	1	0	0	0	0	0	1	0
E4968	0	0	0	0	1	0	0	0	0	0	0	0	0	0	0	0	0	1	0	0	1	0	1
E5246	0	0	0	0	1	0	0	0	1	0	0	0	0	0	0	1	0	0	0	0	0	1	0
E5271	0	0	1	0	1	1	1	0	0	0	0	0	1	0	0	1	0	0	1	0	0	1	0
E5333	0	0	1	0	1	1	1	0	0	0	0	0	1	0	0	1	0	0	1	0	0	1	0
E5369	0	0	1	0	1	1	1	0	0	0	0	0	1	0	0	1	0	0	1	0	0	1	0
E5450	0	1	0	0	1	0	0	0	0	1	1	1	1	0	0	0	0	0	0	0	0	0	0
E5472	0	0	0	0	1	0	0	0	0	0	0	0	0	0	0	1	0	0	0	0	0	1	0
E5476	0	0	1	0	1	1	1	0	0	0	0	0	1	0	0	1	0	0	1	0	0	1	0
E5606	0	0	0	0	1	0	0	0	0	0	0	0	0	0	0	1	0	0	0	0	0	1	0
E5611	0	0	0	0	1	0	0	0	0	0	0	0	0	0	0	1	0	0	0	0	0	1	0
E5879	0	0	0	1	1	0	0	0	0	0	0	0	0	0	0	1	0	0	0	1	0	1	0
E6114	0	0	1	0	1	1	1	0	0	0	0	0	1	0	0	1	0	0	1	0	0	1	0
E6124	0	0	0	0	1	0	0	0	0	0	0	0	0	0	0	1	0	0	0	0	0	1	0
E6199	0	0	0	0	1	0	0	0	0	0	0	0	0	0	0	1	0	0	0	0	0	1	0
E6207	0	0	0	0	1	0	0	0	0	0	0	0	0	0	0	1	0	0	0	0	0	1	0
E6352	0	0	0	0	0	0	0	0	0	0	0	0	1	0	0	1	0	0	0	0	0	1	0
E6439	0	0	0	0	1	0	0	0	0	0	0	0	0	0	0	1	0	0	0	0	0	1	0
E6444	0	0	0	0	1	0	0	0	0	0	0	0	0	0	0	1	0	0	0	0	0	1	0
E6452	0	0	1	0	1	1	1	0	0	0	0	0	1	0	0	1	0	0	1	0	0	1	0
E6488	0	0	1	0	1	1	1	0	0	0	0	0	1	0	0	1	0	0	1	0	0	1	0
E6504	0	0	1	0	1	1	1	0	0	0	0	0	1	0	0	1	0	0	1	0	0	1	0
E6514	0	0	0	0	1	0	0	0	0	0	0	0	0	0	0	1	0	0	0	0	0	1	0
E6605	0	0	0	0	1	0	0	0	0	0	0	0	0	0	0	1	0	0	0	0	0	1	0
E6640	1	1	0	0	1	0	0	0	0	1	1	0	0	0	0	1	0	0	0	0	0	1	0
E6702	0	0	0	0	1	0	0	0	1	0	0	0	0	0	0	1	0	0	0	0	0	1	0
E6718	0	1	0	0	1	0	0	0	0	1	1	1	1	0	0	0	0	0	0	0	0	0	0
E6886	0	0	1	0	1	1	1	0	0	0	0	0	1	0	0	1	0	0	1	0	0	1	0
E6897	0	0	0	0	1	0	0	0	0	0	0	0	0	0	0	1	0	0	0	0	0	1	0
E7334	0	0	0	0	1	0	0	0	0	0	0	0	0	0	0	1	0	0	0	0	0	1	0
E7403	0	1	0	0	1	0	0	0	0	0	0	0	0	0	1	0	0	0	0	0	0	0	0
E7526	0	0	1	0	1	1	1	0	0	0	0	0	1	0	0	1	0	0	1	0	0	1	0

E7567	0	0	1	0	1	1	1	0	0	0	0	0	1	0	0	1	0	0	1	0	0	1	0
E7616	0	0	0	0	1	0	0	0	0	0	0	0	0	0	0	1	0	0	0	0	0	1	0
E7634	0	0	1	0	1	1	1	0	0	0	0	0	1	0	0	1	0	0	1	0	0	1	0
E7657	0	0	0	0	1	0	0	0	0	0	0	0	0	0	0	1	0	0	0	0	0	1	0
E7695	0	0	1	0	1	1	1	0	0	0	0	0	1	0	0	1	0	0	1	0	0	1	0
E7716	0	0	0	0	1	0	0	0	0	0	0	0	0	0	0	1	0	0	0	0	0	1	0
E7750	0	0	0	0	1	0	0	0	0	0	0	0	0	0	0	1	0	0	0	0	0	1	0
E7885	0	1	1	1	0	0	0	0	0	0	0	0	0	0	0	0	1	0	1	1	0	0	0
E7912	0	0	0	0	1	0	0	0	0	0	0	0	0	0	0	1	0	0	0	0	0	1	0
E8036	0	0	1	0	1	1	1	0	0	0	0	0	1	0	0	1	0	0	1	0	0	1	0
E8168	0	0	1	0	1	1	1	0	0	0	0	0	1	0	0	1	0	0	1	0	0	1	0
E8229	0	0	0	0	1	0	0	0	0	0	0	0	0	0	0	1	0	0	0	0	0	1	0
E8300	0	0	0	0	1	0	0	0	0	0	0	0	0	0	0	1	0	0	0	0	0	1	0
E8420	0	0	0	1	0	0	0	0	0	1	1	0	0	0	0	0	1	0	0	1	0	0	0
E8650	0	0	1	0	1	1	1	0	0	0	0	0	1	0	0	1	0	0	1	0	0	1	0
E8772	0	0	1	0	1	1	1	0	0	0	0	0	1	0	0	1	0	0	1	0	0	1	0
E8888	0	0	0	0	1	0	0	0	0	0	0	0	0	0	0	1	0	0	0	0	0	1	0
E8894	0	0	0	0	1	0	0	0	0	0	0	0	0	0	0	1	0	0	0	0	0	1	0
E8996	0	0	1	0	1	1	1	0	0	0	0	0	1	0	0	1	0	0	1	0	0	1	0
E9142	0	0	1	0	1	1	1	0	0	0	0	0	1	0	0	1	0	0	1	0	0	1	0
E9349	0	0	0	0	1	0	0	0	0	0	0	0	0	0	0	1	0	0	0	0	0	1	0
E9490	0	0	1	0	1	1	1	0	0	0	0	0	1	0	0	1	0	0	1	0	0	1	0
E9696	0	0	0	0	1	0	0	0	0	0	0	0	0	0	0	1	0	0	0	0	0	1	0
E9719	0	0	0	0	1	0	0	0	0	0	0	0	0	0	0	1	0	0	0	0	0	1	0
E9733	0	0	1	0	1	1	1	0	0	0	0	0	1	0	0	1	0	0	1	0	0	1	0
G6942	0	0	0	0	1	0	0	0	0	0	0	0	0	0	0	1	0	0	0	0	0	1	0
J3545	0	0	0	0	1	0	0	0	0	0	0	0	0	0	0	1	0	0	0	0	0	1	0
J5817	0	0	0	0	1	0	0	0	0	0	0	0	0	0	0	1	0	0	0	0	0	1	0
J7160	0	0	0	0	1	0	0	0	0	0	0	0	0	0	0	1	0	0	0	0	0	1	0
J9245	0	0	0	0	1	0	0	0	0	0	0	0	0	0	0	1	0	0	0	0	0	1	0
L2400	0	1	0	0	0	0	0	0	0	1	1	0	0	0	0	1	0	0	0	0	0	1	0
L2401	0	1	0	0	0	0	0	0	0	1	1	0	0	0	0	1	0	0	0	0	0	1	0
L2763	0	0	0	0	1	0	0	0	0	0	0	0	0	0	0	1	0	0	0	0	0	1	0
L2814	0	1	0	0	1	0	0	0	0	1	1	1	1	0	0	0	0	0	0	0	0	0	0
L7545	0	0	0	0	1	0	0	0	0	0	0	0	0	0	0	0	0	1	0	0	1	0	1
M2248	0	0	0	0	1	0	0	0	0	0	0	0	0	0	0	1	0	0	0	0	0	1	0
M6600	0	1	0	0	1	0	0	0	1	0	0	0	1	0	0	1	0	0	0	0	0	1	0
M6937	0	0	0	0	1	0	0	0	0	0	0	0	0	0	0	1	0	0	0	0	0	1	0
M7157	0	0	0	0	1	0	0	0	0	0	0	0	0	0	0	1	0	0	0	0	0	1	0
M9253	0	1	0	0	1	0	0	0	1	0	0	0	1	0	0	1	0	0	0	0	0	1	0
M9403	0	0	0	0	1	0	0	0	0	0	0	0	0	0	0	1	0	0	0	0	0	1	0
N8215	0	0	0	0	1	0	0	0	0	0	0	0	0	0	0	1	0	0	0	0	0	1	0
S6670	1	1	0	0	1	0	0	1	0	0	0	0	0	0	1	0	0	0	0	0	0	0	0
X1706	0	0	0	0	1	0	0	0	0	0	0	0	0	0	0	1	0	0	0	0	0	1	0
X2459	0	1	0	0	1	0	0	0	0	0	0	0	0	0	0	1	0	0	0	0	0	1	0
X3426	0	0	0	0	1	0	0	0	0	0	0	0	0	0	0	1	0	0	0	0	0	1	0
X4751	0	0	0	0	1	0	0	0	0	0	0	0	0	0	0	1	0	0	0	0	0	1	0
X4863	0	0	0	0	1	0	0	0	0	0	0	0	0	0	0	1	0	0	0	0	0	1	0

X5705	0	0	1	0	1	1	1	0	0	0	0	0	1	0	0	1	0	0	1	0	0	1	0
X7400	0	0	0	0	0	0	0	0	0	0	0	0	0	0	0	1	0	0	0	0	0	1	0
X7828	0	1	0	0	1	0	0	0	0	0	0	0	0	0	0	1	0	0	0	0	0	1	0
X9177	0	0	0	0	1	0	0	0	0	0	0	0	0	0	0	1	0	0	0	0	0	1	0
X9285	0	0	0	0	1	0	0	0	0	0	0	0	0	0	0	1	0	0	0	0	0	1	0
X9374	0	0	0	0	1	0	0	0	0	0	0	0	0	0	0	1	0	0	0	0	0	1	0
Y1080	0	0	0	0	1	0	0	0	0	0	0	0	0	0	0	1	0	0	0	0	0	1	0
Y1081	0	0	0	0	1	0	0	0	1	0	0	0	0	0	0	1	0	0	0	0	0	1	0
Y1087	0	0	0	0	1	0	0	0	0	0	0	0	0	0	0	1	0	0	0	0	0	1	0
Y1173	0	0	0	0	1	0	0	0	0	0	0	0	0	0	0	1	0	0	0	0	0	1	0
Y1676	0	0	0	0	0	0	0	0	0	0	0	0	0	0	0	0	0	0	0	0	0	0	0
Y1717	0	0	0	0	1	0	0	0	0	0	0	0	0	0	0	1	0	0	0	0	0	1	0
Y1892	0	0	0	0	1	0	0	0	0	0	0	0	0	0	0	1	0	0	0	0	0	1	0
Y1961	0	0	0	0	1	0	0	0	0	0	0	0	0	1	0	1	0	0	0	0	0	1	0
Y2637	0	1	0	0	1	0	0	0	0	0	0	0	0	0	1	0	0	0	0	0	0	0	0
Y2715	0	0	0	0	1	0	0	0	0	0	0	0	0	0	0	1	0	0	0	0	0	1	0
Y2968	0	0	0	0	1	0	0	0	0	0	0	0	0	0	0	1	0	0	0	0	0	1	0
Y3249	0	0	0	0	1	0	0	0	0	0	0	0	0	0	0	1	0	1	0	0	1	1	1
Y3277	0	0	1	0	1	1	1	0	0	0	0	0	0	0	0	1	0	0	1	0	0	1	0
Y3343	0	0	0	0	1	0	0	0	0	0	0	0	0	0	0	1	0	0	0	0	0	1	0
Y3443	0	0	0	0	1	0	0	0	0	0	0	0	0	0	0	1	0	0	0	0	0	1	0
Y3842	0	1	0	0	1	0	0	0	0	0	0	0	0	0	1	0	0	0	0	0	0	0	0
Y4371	0	0	0	0	1	0	0	0	0	0	0	0	0	0	0	1	0	0	0	0	0	1	0
Y4934	0	0	0	0	1	0	0	0	0	0	0	0	0	0	0	1	0	0	0	0	0	1	0
Y4943	1	1	1	0	0	1	0	0	1	1	1	0	0	0	0	1	0	0	1	0	0	1	0
Y5009	1	1	1	0	0	1	0	0	0	1	1	0	0	0	0	1	0	0	1	0	0	1	0
Y5247	0	0	0	0	1	0	0	0	0	0	0	0	0	0	0	1	0	0	0	0	0	1	0
Y5411	0	0	0	1	1	0	0	0	0	0	0	0	0	0	0	1	0	0	0	1	0	1	0
Y5682	0	0	0	0	1	0	0	0	0	0	0	0	0	0	0	1	0	0	0	0	0	1	0
Y5792	0	0	0	0	1	0	0	0	0	0	0	0	0	0	0	1	0	0	0	0	0	1	0
Y5923	0	0	0	0	1	0	0	0	0	0	0	0	0	0	0	1	0	0	0	0	0	1	0
Y6166	0	0	0	0	1	0	0	0	0	0	0	0	0	0	0	1	0	0	0	0	0	1	0
Y6197	0	0	0	0	1	0	0	0	0	0	0	0	0	0	0	1	0	0	0	0	0	1	0
Y6648	0	0	0	0	1	0	0	0	0	0	0	0	0	0	1	1	0	0	0	0	0	1	0
Y6784	0	0	0	0	1	0	0	0	0	0	0	0	0	0	0	1	0	0	0	0	0	1	0
Y7283	0	0	0	0	1	0	0	0	0	0	0	0	0	0	0	1	0	0	0	0	0	1	0
Y7354	0	0	0	0	1	0	0	0	0	0	0	0	0	0	0	1	0	0	0	0	0	1	0
Y7361	0	0	0	0	1	0	0	0	0	0	0	0	0	0	0	1	0	0	0	0	0	1	0
Y7511	0	0	0	0	1	0	0	0	0	0	0	0	0	0	0	1	0	0	0	0	0	1	0
Y7742	0	0	0	0	1	0	0	0	0	0	0	0	0	0	0	1	0	0	0	0	0	1	0
Y7767	0	0	0	0	1	0	0	0	1	0	0	0	0	0	0	1	0	0	0	0	0	1	0
Y8023	0	0	0	0	1	0	0	0	0	0	0	0	0	0	0	1	0	0	0	0	0	1	0
Y8024	0	0	0	0	1	0	0	0	0	0	0	0	0	0	0	1	0	0	0	0	0	1	0
Y8131	0	0	0	0	1	0	0	0	0	0	0	0	0	0	0	1	0	0	0	0	0	1	0
Y8485	0	0	0	0	1	0	0	0	0	0	0	0	0	0	0	1	0	0	0	0	0	1	0
Y8500	0	0	1	0	1	1	1	0	0	0	0	0	1	0	0	1	0	0	1	0	0	1	0
Y8649	0	0	0	0	1	0	0	0	0	0	0	0	0	0	0	1	0	0	0	0	0	1	0
Y8704	0	0	0	0	1	0	0	0	0	0	0	0	0	0	0	1	0	0	0	0	0	1	0

Y9217	0	0	0	0	1	0	0	0	0	0	0	0	0	0	0	1	0	0	0	0	0	1	0
Y9239	0	1	0	0	0	0	0	0	0	0	0	0	0	0	0	1	0	0	0	0	0	1	0
Y9437	0	0	0	0	1	0	0	0	0	0	0	0	0	0	0	1	0	0	0	0	0	1	0
Y9446	0	0	1	0	1	1	1	0	0	0	0	0	1	0	0	1	0	0	1	0	0	1	0
Y9707	0	0	1	0	1	1	0	0	0	1	1	0	0	0	0	1	0	0	1	0	0	1	0
Y9931	0	0	0	0	1	0	0	0	0	0	0	0	0	0	0	1	0	0	0	0	0	1	0
Z5574	0	0	0	0	1	0	0	0	0	0	0	0	0	0	0	1	0	0	0	0	0	1	0
Z7256	0	0	0	0	1	0	0	0	0	0	0	0	0	0	0	1	0	0	0	0	0	1	0
Z7590	0	0	0	0	1	0	0	0	0	0	0	0	0	0	0	1	0	0	0	0	0	1	0
Z9616	0	0	0	0	1	0	0	0	0	0	0	0	0	0	0	1	0	0	0	0	0	1	0
Z9945	0	1	0	0	0	0	0	0	0	0	0	0	0	0	1	1	0	0	0	0	0	1	0

Table S6. The MIC Values and folding points of the 12 antibiotics

Table S6. The MIC Values and folding points of the 12 antibiotics												
Strain name	MIC values of strains (μg/ml)											
	Florfenicol	Cefepime	Levofloxacin	Minocycline	Ampicillin	Penicillin G	Tetracycline	Clindamycin	Enrofloxacin	Erythromycin	Clarithromycin	Daptomycin
C9523	2	0.125	0.25	4	0.125	0.25	16	256	0.25	128	> 128	2
C9726	2	0.125	0.25	8	0.125	0.25	32	> 256	0.25	128	8	2
E1120	1	0.125	0.25	4	0.125	0.25	16	> 256	0.125	128	128	0.5
E1210	1	0.125	0.125	2	0.125	0.125	16	256	0.125	> 128	> 128	1
E1225	16	2	32	8	> 128	> 256	128	256	64	> 128	> 128	> 8
E1529	2	0.125	0.25	8	0.125	0.25	128	256	0.125	> 128	> 128	0.5
E1819	2	0.125	0.25	4	0.125	0.25	16	128	0.125	64	128	0.25
E1974	2	0.125	4	4	0.125	0.5	32	256	2	128	128	1
E1991	2	> 128	32	16	4	4	128	256	64	> 128	> 128	> 8
E2126	2	0.125	0.25	8	0.125	0.25	32	> 256	0.25	128	> 128	0.5
E2128	2	0.125	0.5	16	0.125	0.25	128	> 256	0.125	128	> 128	0.5
E2309	2	0.125	0.5	8	0.125	0.25	32	6	0.25	> 128	> 128	0.5
E2376	1	0.125	0.25	4	0.125	0.25	32	2	0.125	0.125	0.125	0.5
E2498	2	0.125	0.5	8	0.125	0.25	32	128	0.5	128	> 128	1
E2592	2	0.125	0.25	4	0.125	0.25	32	256	0.125	64	> 128	0.5
E2690	2	0.125	0.25	8	0.25	1	32	256	0.25	128	> 128	0.5
E2724	2	0.25	0.25	16	0.125	1	32	> 256	0.125	> 128	> 128	2
E2741	2	0.125	0.25	8	0.25	0.25	32	256	0.25	> 128	> 128	0.5
E2758	16	0.125	0.125	4	0.125	0.25	32	128	0.125	2	0.5	2
E2871	2	0.125	0.25	8	0.125	0.25	16	> 256	0.125	128	> 128	1
E3038	2	0.125	0.25	8	0.125	0.25	32	256	0.125	128	128	1
E3111	16	0.125	8	8	0.125	0.25	32	256	16	2	1	0.5
E3123	1	0.125	0.125	1	0.125	0.25	16	128	0.125	1	0.5	1
E3145	2	> 128	32	16	4	16	128	> 256	32	> 128	> 128	> 8
E3342	16	1	0.5	8	> 128	> 256	32	256	0.125	128	> 128	> 8
E3606	32	0.5	8	4	0.125	0.25	32	> 256	4	> 128	> 128	8

E39 37	32	0.5	8	16	1	2	64	> 256	8	> 128	> 128	1
E39 46	2	0.125	0.25	8	0.125	0.25	32	256	0.125	4	1	0.5
E39 48	2	0.125	1	16	0.5	0.5	128	256	0.5	> 128	> 128	0.5
E40 81	2	0.125	0.125	8	0.125	0.25	32	256	0.125	> 128	> 128	0.5
E42 99	4	0.125	0.25	16	0.125	0.25	32	> 256	0.125	> 128	> 128	4
E43 08	2	0.125	0.25	8	0.125	0.25	64	256	0.125	> 128	> 128	0.5
E43 43	2	0.125	0.25	8	0.125	0.25	32	> 256	0.125	> 128	> 128	0.5
E43 44	2	0.125	0.25	8	0.25	1	32	256	0.25	128	> 128	0.5
E45 04	1	0.125	0.125	8	0.125	0.5	32	256	0.125	1	2	1
E45 13	2	0.125	0.25	4	0.125	0.25	32	256	0.25	> 128	> 128	0.5
E45 55	4	0.125	0.5	16	0.125	0.25	32	> 256	0.25	256	64	0.25
E45 58	4	> 128	32	16	4	1	128	256	64	> 128	> 128	> 8
E48 43	1	0.125	0.25	4	0.125	0.25	32	256	0.125	16	16	4
E49 68	1	0.125	0.25	4	0.125	0.25	32	256	0.125	4	1	0.5
E52 46	16	0.125	0.5	4	0.125	0.25	32	256	0.125	4	1	2
E52 71	2	0.125	0.25	16	0.125	0.25	32	> 256	0.25	128	> 128	0.25
E53 33	2	0.125	0.25	8	0.25	1	32	> 256	0.125	128	> 128	0.5
E53 69	2	0.125	0.5	8	0.125	0.25	64	256	0.25	> 128	> 128	0.5
E54 50	2	0.125	1	16	0.25	0.25	32	256	0.5	> 128	> 128	0.5
E54 72	1	0.125	0.25	8	0.125	0.25	32	> 256	0.125	64	128	0.25
E54 76	64	32	16	8	1	2	128	> 256	16	> 128	> 128	> 8
E56 06	2	0.125	1	0.5	0.125	0.25	16	256	0.125	64	32	0.5
E56 11	32	0.125	0.25	16	0.125	0.25	64	256	0.125	8	2	0.5
E58 79	64	0.125	1	16	0.125	0.5	128	> 256	0.5	128	> 128	2
E61 14	2	0.125	0.25	8	0.25	1	32	256	0.125	128	> 128	0.25
E61 24	1	0.125	0.25	8	0.125	0.25	64	256	0.25	64	8	1
E61 99	2	0.125	0.5	16	0.125	0.125	32	256	0.5	> 128	128	0.5
E62 07	1	0.125	0.25	2	0.125	0.25	16	256	0.25	4	1	0.5
E63 52	1	0.125	0.5	4	0.125	0.25	64	0.125	0.5	0.125	0.125	0.5
E64 39	32	8	16	16	2	2	128	> 256	16	> 128	> 128	> 8
E64 44	2	0.125	0.25	4	0.125	0.25	32	> 256	0.125	> 128	> 128	0.5
E64 52	2	0.125	0.25	8	0.125	0.25	128	256	0.125	128	> 128	0.5
E64 88	2	0.125	0.25	8	0.25	0.5	32	> 256	0.5	128	> 128	1
E65 04	64	0.125	2	8	2	1	32	> 256	8	> 128	> 128	0.5
E65 14	1	0.125	0.5	4	0.125	0.25	64	256	64	> 128	> 128	0.5
E66 05	2	0.125	1	8	0.25	0.25	64	128	0.5	> 128	> 128	0.5
E66 40	16	0.125	0.5	8	0.125	0.25	16	256	0.25	8	2	0.5
E67 02	2	0.125	0.25	8	0.125	0.25	32	256	0.125	> 128	> 128	0.5
E67 18	2	0.062 5	0.5	16	0.125	0.25	64	256	0.5	256	128	0.5
E68 86	2	0.125	0.5	8	0.125	0.5	32	> 256	8	> 128	> 128	0.5

E6897	2	0.125	0.25	8	0.125	0.25	32	> 256	0.125	> 128	> 128	2
E7334	2	0.125	0.25	16	0.125	0.25	32	256	0.25	> 128	> 128	0.25
E7403	16	0.125	16	16	0.25	2	64	256	16	64	> 128	1
E7526	2	0.125	0.5	8	0.25	0.5	32	> 256	0.5	128	128	0.5
E7567	2	0.25	0.25	8	0.5	1	64	> 256	0.125	128	> 128	0.5
E7616	1	0.125	0.25	4	0.125	0.25	32	256	0.125	4	2	1
E7634	32	> 128	32	16	4	8	128	> 256	64	> 128	> 128	> 8
E7657	2	0.125	0.25	8	0.125	0.125	16	> 256	0.125	> 128	> 128	0.5
E7695	64	64	16	8	1	2	64	> 256	16	> 128	> 128	> 8
E7716	1	0.125	0.25	4	0.125	0.125	32	256	0.125	4	1	0.5
E7750	1	0.125	0.25	4	0.125	0.25	8	256	0.125	64	8	1
E7885	32	1	2	16	16	16	32	> 256	2	> 128	> 128	2
E7912	2	0.125	0.5	8	0.125	0.25	64	256	0.125	> 128	> 128	0.5
E8036	2	0.125	4	4	0.125	0.25	32	256	0.125	256	>256	0.5
E8168	2	0.125	0.25	8	0.125	0.5	32	256	0.5	256	>256	0.5
E8229	1	0.125	0.125	4	0.125	0.25	32	256	0.125	4	2	0.5
E8300	2	0.125	0.5	8	0.125	0.25	64	256	0.125	64	32	0.5
E8420	16	0.125	0.5	8	0.125	0.25	32	8	0.25	128	128	1
E8650	2	0.125	0.25	8	0.25	0.5	32	> 256	0.125	128	> 128	0.25
E8772	2	0.125	0.25	8	0.125	0.25	64	256	0.25	> 128	> 128	0.5
E8888	2	0.125	0.5	8	0.125	0.25	64	> 256	0.25	> 128	> 128	0.5
E8894	1	0.125	0.5	4	0.125	0.25	16	256	0.125	> 128	> 128	0.5
E8996	2	0.125	0.25	8	0.25	1	32	> 256	0.25	128	> 128	1
E9142	2	0.125	0.5	8	0.125	0.25	64	256	0.5	> 128	> 128	1
E9349	1	0.125	0.125	8	0.125	0.125	32	256	0.25	1	> 128	1
E9490	> 128	2	8	0.125	0.25	128	> 256	0.125	> 128	> 128	> 128	1
E9696	1	0.125	0.25	4	0.125	0.25	16	256	0.125	16	2	8
E9719	2	0.125	2	16	0.25	0.25	64	> 256	0.5	128	> 128	0.5
E9733	2	0.125	0.5	16	0.125	0.5	64	256	0.25	> 128	> 128	1
G6942	16	> 256	64	32	8	16	256	> 256	32	> 256	> 256	16
J3545	2	0.125	0.5	8	0.125	0.25	32	256	0.25	> 128	> 128	1
J5817	1	0.125	0.5	4	0.125	0.25	32	256	0.25	8	4	2
J7160	2	0.125	0.25	8	0.125	0.25	32	256	0.25	1	4	0.5
J9245	1	0.125	0.5	8	0.125	0.25	32	256	0.5	> 128	> 128	1
L2400	1	0.125	0.25	8	0.125	0.25	16	256	0.25	> 128	> 128	1
L2401	8	0.125	0.25	4	0.125	0.25	32	256	1	> 128	> 128	1
L2763	1	0.125	0.25	4	0.25	0.25	32	256	0.125	16	4	0.5
L2814	2	0.5	0.5	4	0.125	0.5	64	128	0.125	128	128	0.5
L7545	16	0.125	0.25	2	0.125	0.25	32	256	0.125	2	1	1
M2248	64	8	16	16	2	2	128	256	16	> 128	> 128	> 8

M6600	2	0.125	0.5	8	0.125	0.25	128	256	0.125	> 128	> 128	1
M6937	2	0.125	0.5	2	0.125	0.25	32	> 256	0.25	128	> 128	0.5
M7157	2	0.125	0.25	16	0.125	0.25	32	256	0.25	2	> 128	0.5
M9253	128	16	1	16	> 128	> 256	256	> 256	0.125	128	> 128	> 8
M9403	8	0.125	0.25	2	0.125	0.25	16	> 256	0.125	32	8	0.25
N8215	4	> 128	32	16	8	16	128	> 256	128	> 128	> 128	> 8
S6670	4	0.125	0.5	8	0.125	0.25	32	> 256	0.125	128	> 128	1
X1706	1	0.125	0.25	4	0.125	0.25	32	> 256	0.125	> 128	> 128	0.5
X2459	1	0.125	0.25	4	0.125	0.25	32	256	0.125	32	8	0.5
X3426	2	0.125	0.25	8	0.125	0.25	32	256	0.125	> 128	128	0.5
X4751	1	0.125	0.25	8	0.5	0.25	32	256	0.5	32	128	0.25
X4863	2	0.125	0.5	8	0.125	0.25	64	> 256	0.125	> 128	> 128	0.5
X5705	2	0.125	0.5	8	0.125	0.25	32	256	0.5	128	> 128	0.5
X7400	2	1	0.25	8	0.125	0.25	32	256	0.5	128	> 128	1
X7828	1	0.125	0.25	4	0.125	0.25	32	256	0.25	128	128	0.5
X9177	2	0.125	0.25	8	0.125	0.25	32	256	0.25	128	> 128	0.5
X9285	2	0.25	0.125	4	0.125	0.25	16	128	0.125	4	1	2
X9374	2	0.125	0.5	8	0.125	0.25	32	256	0.25	64	128	0.25
Y1080	1	0.125	0.25	4	0.125	0.25	16	256	0.25	128	128	0.5
Y1081	2	0.125	0.25	8	0.125	0.25	32	256	0.25	> 128	> 128	0.5
Y1087	32	0.125	0.25	16	0.125	0.25	16	256	0.125	32	32	0.5
Y1173	2	0.125	0.5	8	0.125	0.5	32	256	0.25	128	> 128	0.5
Y1676	2	0.125	0.25	1	0.125	0.25	16	256	0.25	4	1	0.5
Y1717	16	0.125	8	8	0.125	0.25	32	256	16	2	1	0.5
Y1892	2	0.125	0.25	8	0.125	0.25	32	256	0.25	128	> 128	0.5
Y1961	64	0.125	0.25	8	0.125	0.5	64	> 256	0.125	> 128	> 128	0.5
Y2637	1	0.125	0.5	8	0.125	1	64	256	1	> 128	> 128	2
Y2715	2	0.25	0.25	16	0.125	0.125	32	> 128	0.5	32	> 128	0.5
Y2968	1	0.125	2	16	0.25	0.25	16	16	2	> 128	> 128	0.25
Y3249	2	0.125	0.125	16	0.125	0.125	32	> 128	0.125	8	4	0.5
Y3277	2	0.125	0.125	8	0.125	0.125	> 128	> 128	0.25	8	> 128	0.25
Y3343	1	0.125	0.5	4	0.125	0.25	32	256	0.125	4	2	0.5
Y3443	2	0.125	0.25	8	0.125	0.25	32	256	0.25	> 128	> 128	0.5
Y3842	2	0.125	0.5	8	0.125	0.25	32	256	0.25	128	128	0.5
Y4371	16	0.125	0.125	8	0.125	0.25	32	256	0.5	64	8	1
Y4934	2	0.125	0.25	8	0.125	0.125	32	256	0.25	8	4	0.5
Y4943	16	0.125	8	16	0.125	1	32	256	16	> 128	> 128	0.5
Y5009	16	0.125	8	4	0.5	0.25	32	256	8	8	2	1
Y5247	2	0.125	0.25	8	0.125	0.125	32	256	0.5	256	>256	1
Y5411	32	0.125	0.25	16	0.125	0.25	32	256	0.25	2	1	0.5

Y5682	2	0.125	0.25	8	0.125	32	32	> 128	0.25	32	> 128	0.5
Y5792	2	0.125	0.25	0.5	0.125	0.25	16	> 256	0.25	> 128	> 128	1
Y5923	2	0.125	0.25	8	0.125	0.25	32	256	0.125	> 128	> 128	1
Y6166	2	16	0.5	16	2	1	32	256	0.25	> 128	> 128	32
Y6197	2	0.125	1	8	0.25	0.5	64	128	0.5	> 128	> 128	0.5
Y6648	1	0.125	0.125	8	0.125	0.25	32	0.25	0.125	1	0.125	0.25
Y6784	2	0.125	0.5	8	0.125	0.25	128	256	0.125	> 128	> 128	0.5
Y7283	1	0.125	0.25	4	0.125	0.25	32	256	0.125	> 128	> 128	2
Y7354	2	0.125	0.25	4	0.125	0.125	16	256	0.125	16	4	1
Y7361	2	0.125	0.25	16	0.125	0.25	64	> 256	0.125	128	128	1
Y7511	2	0.125	0.25	8	0.125	0.25	64	256	0.25	64	128	1
Y7742	32	16	0.5	16	> 128	> 256	128	> 256	0.5	> 128	> 128	> 8
Y7767	2	0.125	0.25	8	0.125	0.25	32	256	0.125	0.25	> 128	0.5
Y8023	2	0.125	1	8	0.125	0.25	64	256	0.25	64	64	1
Y8024	2	0.125	0.25	16	0.25	0.25	32	> 256	0.125	128	> 128	2
Y8131	1	0.125	0.25	4	0.125	0.125	32	256	0.125	128	128	0.5
Y8485	2	0.125	0.5	8	0.125	0.25	32	256	0.25	16	32	0.5
Y8500	1	0.125	0.25	8	0.125	0.25	32	256	0.25	128	> 128	0.5
Y8649	4	> 128	32	16	2	8	128	256	32	> 128	> 128	> 8
Y8704	2	0.125	0.5	8	0.125	0.25	32	256	0.125	128	128	0.5
Y9217	2	0.125	0.25	8	0.125	0.25	32	256	0.25	16	16	0.5
Y9239	32	> 128	4	32	8	16	128	> 256	4	> 128	> 128	> 8
Y9437	2	0.125	0.25	0.25	0.125	0.25	32	> 256	0.25	> 128	> 128	0.5
Y9446	1	0.125	32	16	0.125	0.25	32	128	32	> 128	> 128	0.5
Y9707	1	0.125	8	8	0.125	0.125	32	64	16	128	128	1
Y9931	2	0.125	0.25	8	0.125	0.25	8	256	0.5	8	4	0.5
Z5574	2	0.125	1	8	0.25	0.25	64	64	2	> 128	> 128	0.25
Z7256	2	0.125	8	16	0.125	0.5	32	> 256	2	> 128	> 128	0.5
Z7590	2	0.125	0.25	16	0.0625	0.125	64	256	0.5	256	>256	1
Z9616	2	0.125	0.5	8	0.125	0.25	16	128	0.5	> 128	> 128	0.25
Z9945	8	0.125	0.25	8	0.125	0.25	32	256	0.125	> 128	> 128	0.5
E7912	2	0.125	0.5	8	0.125	0.25	64	256	0.125	> 128	> 128	0.5

Table S7. The data of the BALB/c mice infection model

**Table S7. The data of the BALB/c mice infection model**							
Strain name	Number of deaths of mice during infection (n=5)					Clade	VF Cluster
	12h	24h	48h	72h	108h		
E7634	0	4	4	4	4	HAC	II
E5476	0	3	4	5	5	HAC	II
E7695	0	0	0	0	0	HAC	II
Y9437	0	2	2	2	2	HAC	II

E7885	0	3	5	5	5	HPC	I
E7403	0	3	5	5	5	HPC	I
E1991	2	5	5	5	5	HPC	I
Z7590	0	5	5	5	5	HPC	I
Y8023	5	5	5	5	5	HPC	I
E5879	0	3	3	3	3	HPC	III
E3606	0	2	3	3	3	HPC	III
G6942	0	2	2	2	2	HPC	III
Y9707	0	2	2	2	2	HPC	III
E3111	0	0	0	0	0	HPC	IV
Y7742	2	5	5	5	5	DPC	III
E3145	4	5	5	5	5	DPC	III
E6207	0	3	4	4	4	DPC	III
E6199	0	2	3	4	4	unknown	IV
Z7256	0	1	2	2	2	unknown	IV
E5611	0	1	1	1	1	unknown	IV
SC19	1	5	5	5	100	/	/
